# Cost-free charity facilitates dishonesty in a dice-rolling experiment

**DOI:** 10.1038/s41598-026-56788-9

**Published:** 2026-06-19

**Authors:** Judit Mokos, Anna Fedor, Dorottya Deli, Borbála Kívés, Zsóka Vásárhelyi, Gergely Boza, István Scheuring

**Affiliations:** 1 Department of Plant Taxonomy, Ecology and Theoretical Biology, Eötvös University, Budapest, Hungary; 2 Institute of Evolution, HUN-REN Centre for Ecological Research, Budapest, Hungary; 3 Institute of Ecology and Botany, HUN-REN Centre for Ecological Research, Budapest, Hungary; 4 Cooperation and Transformative Governance Research Group and Exploratory Modeling of Human-natural Systems Research Group, Advancing Systems Analysis Program, International Institute for Applied Systems Analysis (IIASA), Laxenburg, Austria

**Keywords:** Mathematics and computing, Psychology, Psychology

## Abstract

Exploring the motivations behind collaboratively dishonest behaviour, is often done using dice-rolling experiments, where coordinated cheating increases participant pairs’ earnings. Here we present a preregistered dice-rolling experiment that investigates how participants’ dishonest (cheating) behaviour is influenced (i) by the dishonest (cheating) behaviour of their experimental partners and (ii) by by-product altruism (or cost-free charity) that is a direct consequence of a monetary reward in this experiment. We studied a 2 × 2 factorial design of the dice-rolling game, with the presence or absence of (i) a cheating partner and (ii) by-product altruism. Following the game, participants filled out the Moral Foundations Questionnaire and the Social Dominance Orientation questionnaire. We found that without the opportunity of cost-free charity, cheating was not detectable, independent of the cheating behaviour of the partner. However, the opportunity of donating to a chosen charitable foundation (cost-free charity) significantly increased the level of cheating. We found that the relationship between the degree of dishonesty and the measured psychological traits is complex and, in some cases, contradictory. Our results do, however, confirm that participants with a stronger moral integrity were less likely to cheat. Our results showed that the level of collaborative cheating to obtain a monetary benefit is significantly increased by participants’ perception of acting in a socially beneficial way as a by-product of their unethical behaviour.

**Supplementary Information:**

The online version contains supplementary material available at https://doi.org/10.1038/s41598-026-56788-9.

## Introduction

### Background

The healthy functioning of human societies is founded on the pro-social behaviour of their members^1^. These include various forms of cooperation and collective actions^2^, maintained by social norms and institutions, and by the sanctioning or punishing of rule violators^3,4^. In contrast, dishonest behaviour, such as deception or cheating, deeply undermines trust in institutions, and disrupts interpersonal interactions^5-7^. It thus undermines cooperation in general and self-reporting regimes, such as filling tax returns or insurance claims^8,9^.

Thoughtfully designed experiments can uncover the mechanisms behind dishonest or cheating behaviour in humans^7,10,11–14^. Among these experiments, the so-called dice-rolling experiments play a prominent role. In a pioneering dice-rolling experiment by Fischbacher and Föllmi-Heusi (to which we will later refer to as the FF experiment)^15^, participants rolled a die in private and then reported the results. They won units of money equal to the reported number, except when it was six, in which case they did not win. Cheating was indicated by the fact that the reported values differed significantly from the uniform distribution that characterises the distribution of numbers rolled randomly on dice. In this experiment numbers six and one were significantly underrepresented, while numbers four and five were overrepresented, indicating that participants were willing to lie for a higher reward. A meta-analysis of such experiments concluded that the rate of cheating is typically not high^16^, measured by the participants obtaining roughly a quarter of the theoretically highest income with the maximum levels of cheating. Honesty is strongly preferred, even in these anonymous experiments^16^.

Lewis et al.^17^ modified the FF experiment so that the announced result of the roll of the die did not generate income for the player, but was instead a donation to Cancer Research UK. In absence of a control setting, these results were compared to the original FF experiment, and the authors argued that participants’ incentives for donation encouraged dishonesty more than did the prospect of personal gain. By contrast, a more recent paper found that cheating was less prevalent if the monetary reward went to another anonymous participant instead of the decision maker^18^. Using the FF experimental framework, Jacobsen and Piovesan^9^ studied another type of incentive for dishonesty. They compared the situations of the monetary reward being tax-free or taxed. They observed that dishonesty was higher in their experimental group where they introduced a tax compared to the tax-free control^9^. In another variant of the FF experiment^19^, it was examined how individuals’ propensity for dishonesty changes if online anonymous communication between the individuals is allowed before recording the number rolled^19^. They showed that communication rapidly and strongly pushed individuals toward increased cheating, despite the the use of the chat being completely optional and its content being created all by the participants. Interestingly, the level of dishonesty in a similar FF experiment decreases with the proximity of a holy day for religious participants^20^. Meanwhile, the greater the elapsed time between the experiment and receiving the reward, the lower the level of cheating in another, similar FF experimental setup^21^.

These follow-up studies organised around Fischbacher and Föllmi-Heussi’s experimental setup^15^ clearly show that the willingness to engage in dishonest behavior is highly dependent on a number of different circumstances. The experiments suggest that beside a direct economic interest^9,21^, cheating is driven by supporting a socially accepted goal^17^, but not by supporting strangers^18^, despite the fact that pro-social cheating typically reduces feelings of guilt^10,22,23^. Other studies of those presented above demonstrate that the extent of cheating depends on the moral integrity of the cheaters, which can be strengthened to a certain extent by religious norms^20^, but can also be quickly and significantly weakened by communication between participants^19^.

The present work is based on another version of the FF experiment, developed by Weisel and Shalvi^12^. Weisel and Shalvi’s experimental setup (WS experiment from now on) contained a collaborative variant of the dice rolling experiment, which revealed, to use the authors’ phrase, “the dark side of cooperation: corrupt collaboration”. In their setup, players were paired up (though could not see each other), privately rolled their dice and reported their values sequentially to each other. They won money only if both of them reported the same value. Every pair played 20 rounds. At the end of the game, the computer randomly selected one round and both participants were paid according to the score of this single round. Each of them earned the reported numbers in euros (e.g., if both reported 5, each received 5 EUR). In this setup, the player who rolled second (Player B) could decide about winning money for both of them by reporting the same number as Player A, even if it was a lie. They found that the reported values deviated to an extreme degree from a uniform distribution, and this was far greater in the collaborative situation than if participants were playing just for Players themselves. We note that Wouda et al.^24^ replicated the “aligned outcomes” condition from Weisel and Shalvi’s experiment^12^ and they received qualitatively similar results, though the measured effect was quantitatively smaller. They attributed this partly to differences between the participant pools: their participants had backgrounds in psychological studies, while Weisel and Shalvi’s participants had backgrounds in economic studies. A subsequent meta-analysis also showed that in collaborative dishonesty experiments, dishonesty increases when the amount of the reward is increased, when there are more men than women in the experiment, and when there are more young than older participants^25^.

The experiments described above aim to uncover, under controlled conditions, which factors influence dishonest and morally unacceptable behaviours that benefit participants, or more concretely, to identify which external social conditions and/or internal personality traits facilitate or hinder dishonest behaviour. These questions have been studied in detail in the context of the non-collaborative FF experiment, as presented above; they have, however, received less attention in the context of the collaborative WS experiment. This situation warrants further attention, especially as Weisel and Shalvi^12^ found that a cooperative setup was tended to increase the likelihood of cheating greatly.

Here we present a modified WS experiment with a 2 × 2 factorial design, in which we study how dishonesty is affected by the presence or absence of (i) a dishonest (cheating) experimental partner and (ii) by-product altruism that is as a direct consequence of a monetary reward in this experiment.

## Hypotheses and design

### Moral erosion and by-product altruism

We hypothesise that the increased level of dishonest behaviour in the WS experiment has two different causes: moral erosion and by-product altruism. Moral erosion happens when the participants realise that their partners are immoral (cheaters), and thus it becomes morally more acceptable for them to cheat, too. In what follows, this will be called the *moral erosion hypothesis*. To test the moral erosion hypothesis, we measure whether the more the partner cheats, the more likely it is that the other person in the pair will also cheat. In the WS experiment, by-product altruism means that when Player B wins a reward, she/he automatically and increases the reward of Player A, too, cost-free. Thus in this case, dishonest behaviour has an additional purpose: helping others without a financial cost. Socially rewarded altruistic behaviour has a strong positive effect on self-esteem^26,27^ and thus adds an additional justification for dishonest behaviour in this situation^13^. Thus, altruism with socially acceptable by-products is likely to increase the temptation to cheat. In what follows, this is called the *charity hypothesis* in the following. To test the charity hypothesis, we measure whether the opportunity to donate to a charity cost-free increases the dishonesty of participants.

To tease out these effects within the WS experimental framework, we planned a series of experiments with a 2 × 2 factorial design. The game was similar to Wiesel and Shalvi’s study^12^, in which participants played in pairs in the dice-rolling experiment, and could win only if they reported rolling the same value. Our participants were always assigned the role of Player B. Because they observed Player A’s reported value before reporting their own, they could influence whether the pair received a reward through deliberate deception or dishonesty. Although Player A was simulated by the computer, participants were told that they were playing with a real person located remotely at another university. Player A was either Honest (its reported values were sampled from a uniform distribution) or Dishonest (higher values were sampled with higher frequency) to test the moral erosion hypothesis. To test the charity hypothesis, half of the participants had to choose a charity foundation which would receive a small donation if they won (we call this the Charity game). The other half of the participants played the original version of the game with no donation (we call this the Simple game).

These two factors produce four conditions in a two-by-two design:

- SH: Simple game with Honest partner.
- SD: Simple game with Dishonest partner.
- CH: Charity game with Honest partner.
- CD: Charity game with Dishonest partner.

The outcome allows us to note whether the distribution of reported doubles deviates from uniformity across the four conditions, in which case, a higher incidence of doubles indicates dishonesty. We tested the moral erosion hypothesis by comparing the number of doubles in conditions with Honest vs. Dishonest partners (SH-SD and CH-CD comparisons) and by examining whether dishonesty increases with the number of rounds, depending on the honesty of the partner. We tested the charity hypothesis by comparing the conditions with the Charity game vs. the Simple game (SH-CH and SD-CD comparisons). We predicted that both the presence of charity and a dishonest partner would increase player dishonesty. We did not have any expectations as to which one of these effects would be stronger.

Sample sizes and statistical tests regarding these hypotheses were pre-registered (see Disclosures).

### Personality and cheating

Apart from the pre-registered hypotheses and analyses mentioned above, we also performed exploratory data analysis to identify possible associations between certain participant personality traits and propensity for cheating across the different experimental setups.

Evidence suggests that certain moral domains influence performance and behaviour in economic exchange games^28^, thus we asked whether cheating in our experiment was also influenced by moral domains. The *Moral Foundations questionnaire* is a reliable tool to measure individual differences in five main moral domains: Harm/care, Fairness/reciprocity, Ingroup/loyalty, Authority/respect, and Purity/sanctity^29^. We used the validated Hungarian version of this questionnaire^30^ to test our expectations:

- We did not expect the moral domain *Harm/care* to be associated with cheating per se. However, as the potential donation in the Charity setting could evoke helping behaviour, we expected people with high scores along this domain to cheat more in the Charity settings (CH, CD).
- We expected the moral domain *Fairness/reciprocity* to be associated with less cheating, as people who rank high in fairness would be less likely to violate the rules.
- We expected the moral domain *Ingroup/loyalty* to be associated with a greater propensity for cheating, especially with a dishonest partner (conditions SD and CD).
- We expected the moral domain *Authority/respect* to be associated with less cheating, due to the respect for the rules of the game.
- We did not expect the moral domain *Purity/sanctity* to be associated with cheating.

According to the Social Dominance Theory, most contemporary human societies are organised as group-based social hierarchies^31^. The *Social Dominance Orientation Questionnaire* is a tool to measure individual differences in how we think about social domination and power^32^. The questionnaire was originally designed to tackle one factor, but later a bifactorial model proved capable of describing social dominance orientation better^32–34^. One subfactor is the SDO-Dominance, which is associated with the support of group-based dominance, even if it includes oppressive or violent acts. The other is the SDO-Anti-egalitarianism, which is associated with a preference for inequality, and an aversion towards the egalitarian redistribution of resources^32^. For these reasons.

- We hypothesised that a social dominance orientation in general would be associated with a higher propensity for cheating.
- Furthermore, we predicted that people ranked higher in each of the subfactors will not show a greater propensity for cheating in the Charity condition. The reason for this is that these people tend to be less keen on helping those in need, therefore we expected that the charity opportunity would not affect their behaviour.

## Results

In this study, we performed a modified version of Weisel and Shalvi’s experiment^12^ with introducing (i) a dishonest partner and (ii) an opportunity for by-product altruism in a 2 × 2 factorial design. The resulting four experimental conditions are designated Simple Honest (as a control), Simple Dishonest, Charity Honest, and Charity Dishonest. For a detailed description of the experiment, see the Methods.

### The distribution of reported numbers

Rolling an unloadeddie produces uniformly distributed numbers between one and six. Deviating from the uniform distribution when reporting the rolled values indicates cheating, but at the same time, a uniform distribution of the reported numbers does not necessarily mean that participants did not cheat. In the case of a completely honest partner who reports uniformly distributed numbers, participants may cheat by matching these numbers to produce doubles, thus, in the process reporting uniformly distributed numbers, too. First, we will describe the distribution of self-reported values across the four experimental setups.

**Fig. 1:**
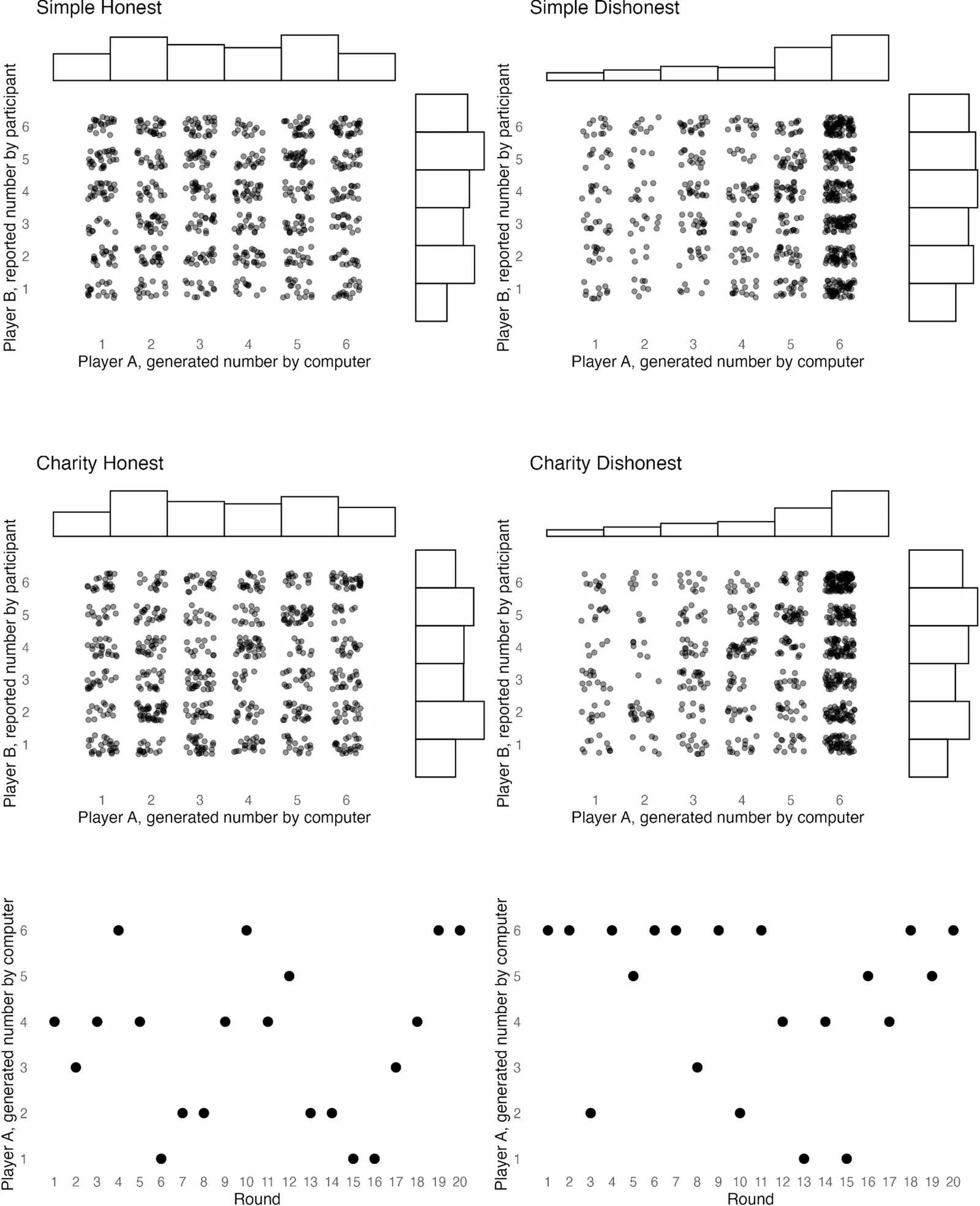
The reported value pairs in the four conditions. The doubles that might win money are located on the main diagonal. Each dot represents one round of the game. Dots are transparent and jittered to decrease overlapping. At the top and right of the graphs, you can see the distribution of reported (Player B) and computer-generated (Player A) rolls. The two figures in the bottom row each show an example of a series of computer-generated numbers for the honest (left) and dishonest (right) settings.

The distribution of self-reported values in Dishonest conditions (SD and CD) is significantly different from the uniform distribution (SD: Chi-squared test $\:\chi\:$^2^=11.2, *p* = 0.036; CD: $\:\chi\:$ ^2^=14.4, *p* = 0.011). Similarly, in the Honest condition without the opportunity of charity (SH), the distribution of the reported numbers differs from the uniform distribution (Chi-squared test, $\:\chi\:$ ^2^=14.7, *p* = 0.012). In contrast, the distribution of reported numbers did not differ significantly from the uniform distribution in the Charity Honest (CH) condition (Chi-squared test, $\:\chi\:$ ^2^=3.3, *p* = 0.67) (for distributions see Table S1 in the Supporting information). However, as we noted above, these results do not reliably inform us whether participants cheated or not (Fig. 1).

As shown in the two upper left panels of Fig. 1, in the case of an honest partner, values 2 and 5 occur slightly more frequently among the computer-generated values, which is reflected in the distribution of participant-reported values, implying that cheating may have occurred during the game. This would be similarly indicated if there were visibly more points in the main diagonal, but this cannot be detected at a glance. In the case of a dishonest partner, the distribution of reported values does not visibly follow the distribution of the partner’s throws, although values of 4–6 are apparently more frequent, and the distribution is not uniform, as we have found. The two lower figures in Fig. 1 illustrate the typical rolled values experienced by a participant when their partner was honest (lower left figure) and dishonest (lower right figure).

### The number of doubles

**Fig. 2:**
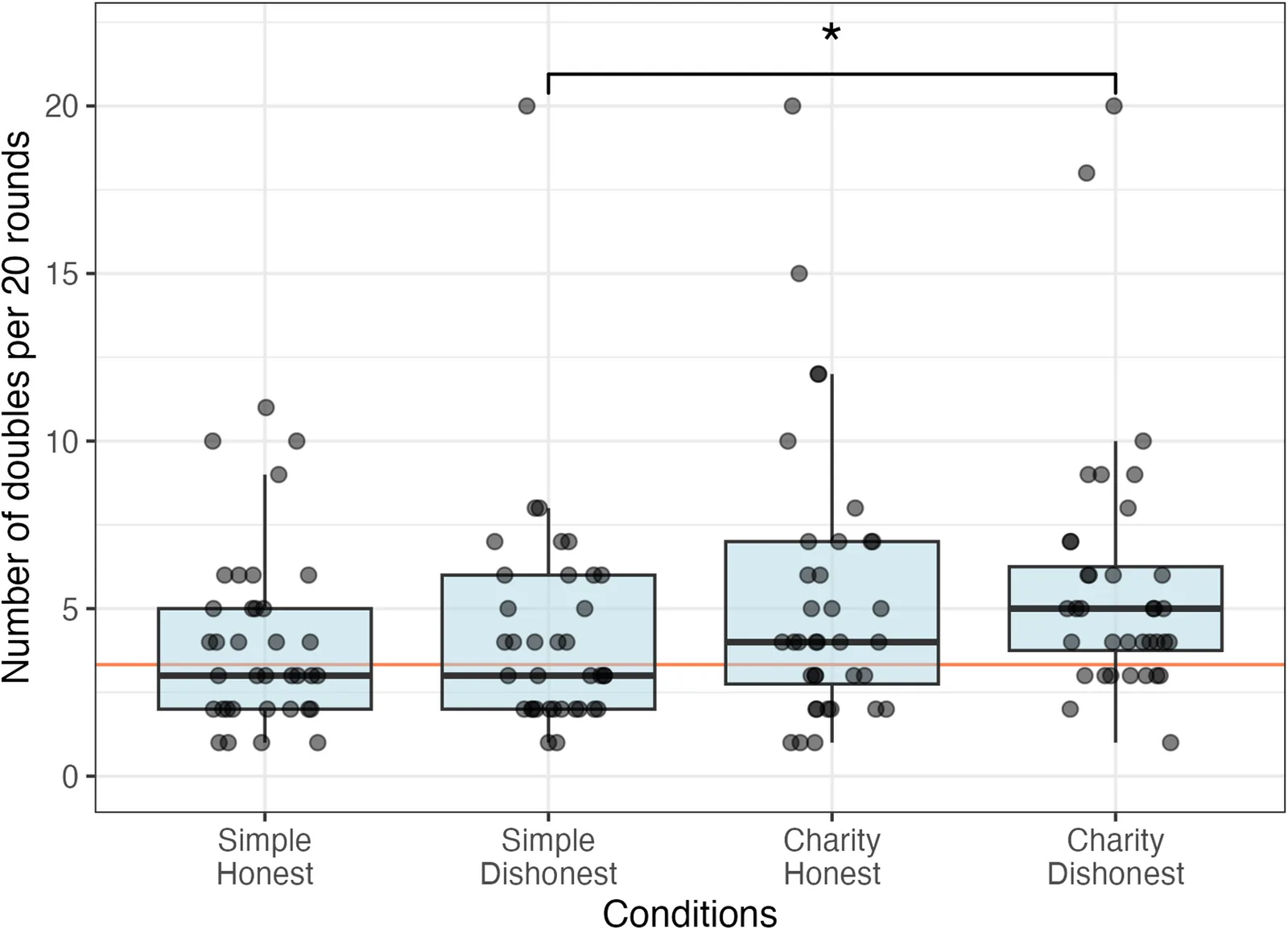
The number of doubles in the four conditions. Data points are transparent and horizontally jittered to avoid overlap. The orange line indicates the expected number of doubles in the case of an unloaded die (3 ⅓ from 20 pairs of rolls). The asterisk represents a significant difference.

There were significantly more reported doubles than the expected value of 20 × 1/6 = 3 1/3 in both Charity conditions. (CH: one-sided one-sample Wilcoxon signed-rank U test, *W* = 489, *p* = 0.007, median = 4; CD: one-sided one-sample Wilcoxon signed-rank U test, *W* = 599, *p* < 0.001, median = 5, ). The value of the same difference was not significant in conditions without the donation opportunity (SH: one-sided one-sample Wilcoxon signed-rank U test, *W* = 400, *p* = 0.147, median = 3; SD: one-sided one-sample Wilcoxon signed-rank U test, *W* = 407, *p* = 0.123, median = 3) (Fig. 2). In each of the three conditions SD, CH and CD, there was one participant who reported the same number as the computer every time (see the dots on the uppermost line of Fig. 2).

When comparing the two Simple conditions to each other, there was no difference between the number of doubles (SH vs. SD: one-sided two-sample Mann-Whitney U test, *W* = 635.5, *p* = 0.445), and similarly, when comparing the two Charity conditions to each other, there was no difference between the number of doubles (CH vs. CD: one-sided two-sample Mann-Whitney U test, *W* = 551.5, *p* = 0.138), indicating that a dishonest partner did not increase cheating. A similar set of comparisons can unravel the role of the charity opportunity: comparing SH to CH showed no significant difference (one-sided two-sample Mann-Whitney U test, *W* = 538, *p* = 0.107), but SD vs. CD differed significantly (one-sided two-sample Mann-Whitney U test, *W* = 434, *p* = 0.008, Fig. 2). The Kruskall-Wallis test confirms the results of pairwise comparisons by showing that the four conditions are significantly different (χ^2^ = 8.080, df = 3, *p* = 0.043). These findings indicate that the opportunity for by-product altruism plays a more important role than the honesty of the partner when the decision of whether to cheat or not is made.

This result is supported by the linear model investigating the effect of by-product altruism and the honesty of the partner on the number of reported doubles: only the Charity setting showed a significant effect on increasing the number of reported doubles (Table 1).

**Table 1:** The effect of charity, partner honesty and their interaction on the number of reported doubles, Poisson regression.

(Intercept)	Estimate	Std. Error	z value	*p* value
	1.749	0.069	25.167	< 0.001
Game (simple)	-0.302	0.106	-2.835	**0.004**
Partner (honest)	-0.085	0.100	-0.853	0.393
Game * Partner	0.031	0.153	0.209	0.834

**Fig. 3:**
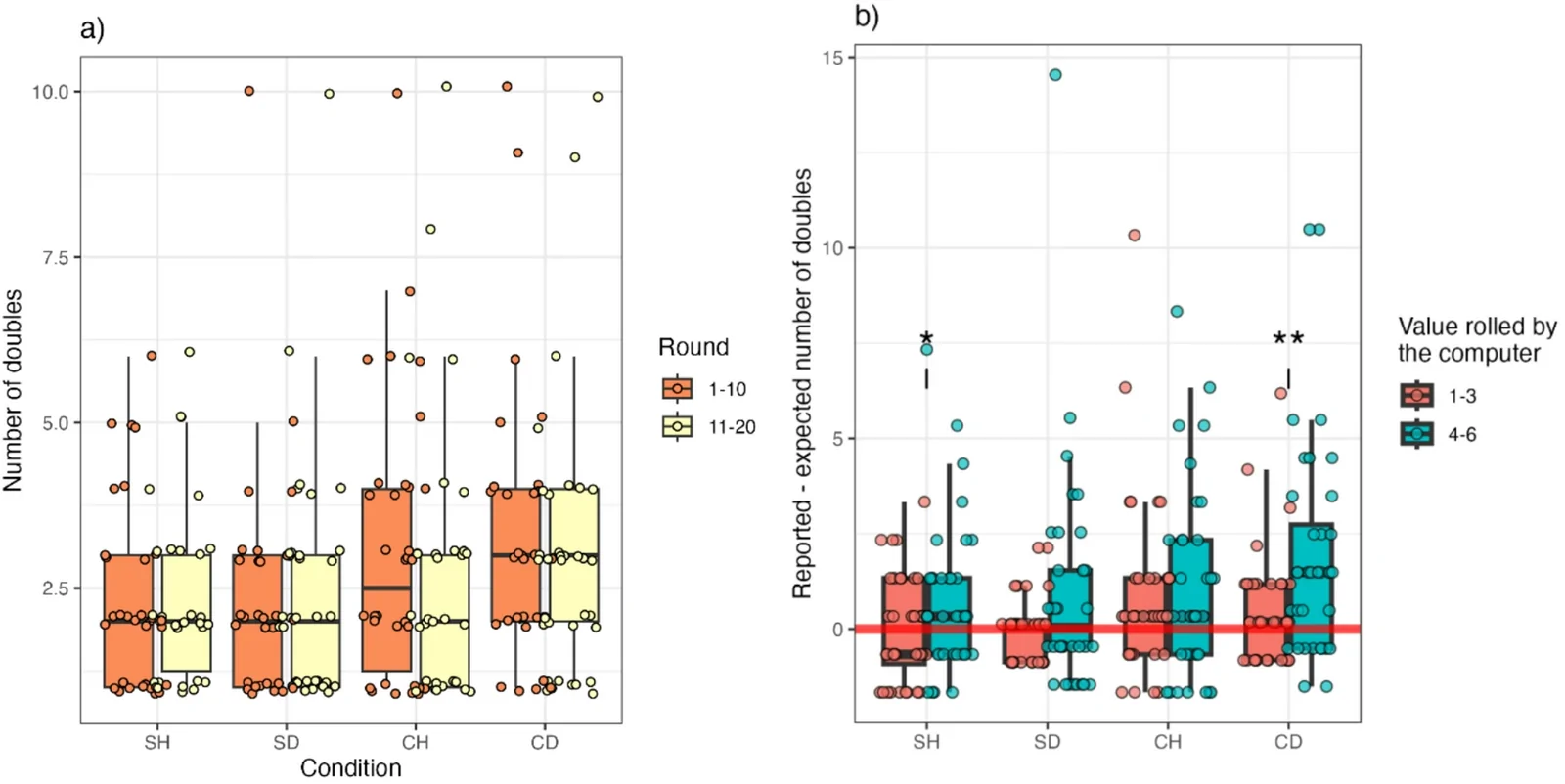
Changes in the propensity to cheat during the experiment and as a function of the number rolled by Player A. **a**) The number of doubles reported in the first and second 10 rounds in the four conditions. **b**) The difference between the number of reported and expected doubles compared to the responses given for numbers 1, 2, 3 and 4, 5, 6 by Player A. The horizontal line at zero shows no difference between the reported and expected number of doubles. Datapoints are transparent and jittered to avoid overlapping datapoints (*= *p* < 0.05, **=*p* < 0.01).

To test for moral erosion, we examined whether participants were more (or less) likely to report doubles at the end of the series of die rolls than at the beginning. We used a series of binomial regressions to study whether there is a relationship between the reported doubles and the number of rounds. No significant relationships were found (Supporting information, Table S4). Thus, we used a more sensitive test to detect moral erosion by comparing the number of doubles reported in the first 10 die rolls with those reported in the second 10 die rolls in any of the settings, but we did not find significant differences between the first and second parts of the game (Fig. 3a).

We also examined whether larger potential winnings, that is, doubling higher numbers, were more tempting and therefore more likely to elicit cheating. Specifically, we assumed that participants might respond differently to rolls of 1–2–3 than to rolls of 4–5–6. To test this, we calculated, for each participant, the difference between the expected and the actual number of reported doubles. This was done separately for responses to machine rolls of 1–2–3 and 4–5–6. In the Honest conditions, the expected number of doubles was 1⅔ for both cases. In the Dishonest conditions, expected values were calculated based on the observed distributions in those conditions (see Supporting Information, Table S3). The resulting expected values were: 0.870 for SD 1–2–3; 2.463 for SD 4–5–6; 0.819 for CD 1–2–3; and 2.514 for CD 4–5–6. We then used a paired Mann–Whitney test to compare, within participants, the difference between expected and actual doubles when responding to low rolls (1–2–3) versus high rolls (4–5–6). As shown in Fig. 3b, participants in the CD and SH conditions reported significantly more doubles in response to high rolls (4–5–6) than to low rolls (1–2–3) (CD: *V* = 15.5, *p* = 0.002; SH: *V* = 109, *p* = 0.03). No significant differences were found in the remaining conditions (SD: *V* = 272, *p* = 0.340; CH: *V* = 227.5, *p* = 0.692).

### Dishonesty and psychological traits

To shed light on the relationship between psychological traits and experimental outcomes, we calculated the Spearman correlation between the number of reported doubles and the subscales of the Moral Foundation Questionnaire (MFQ) and the Social Dominance Orientation questionnaire (SDO7) separately for the four conditions. Although this process revealed several significant correlations, after alpha correction, all correlations disappeared (See Methods and Supporting information Table S5).

**Fig. 4:**
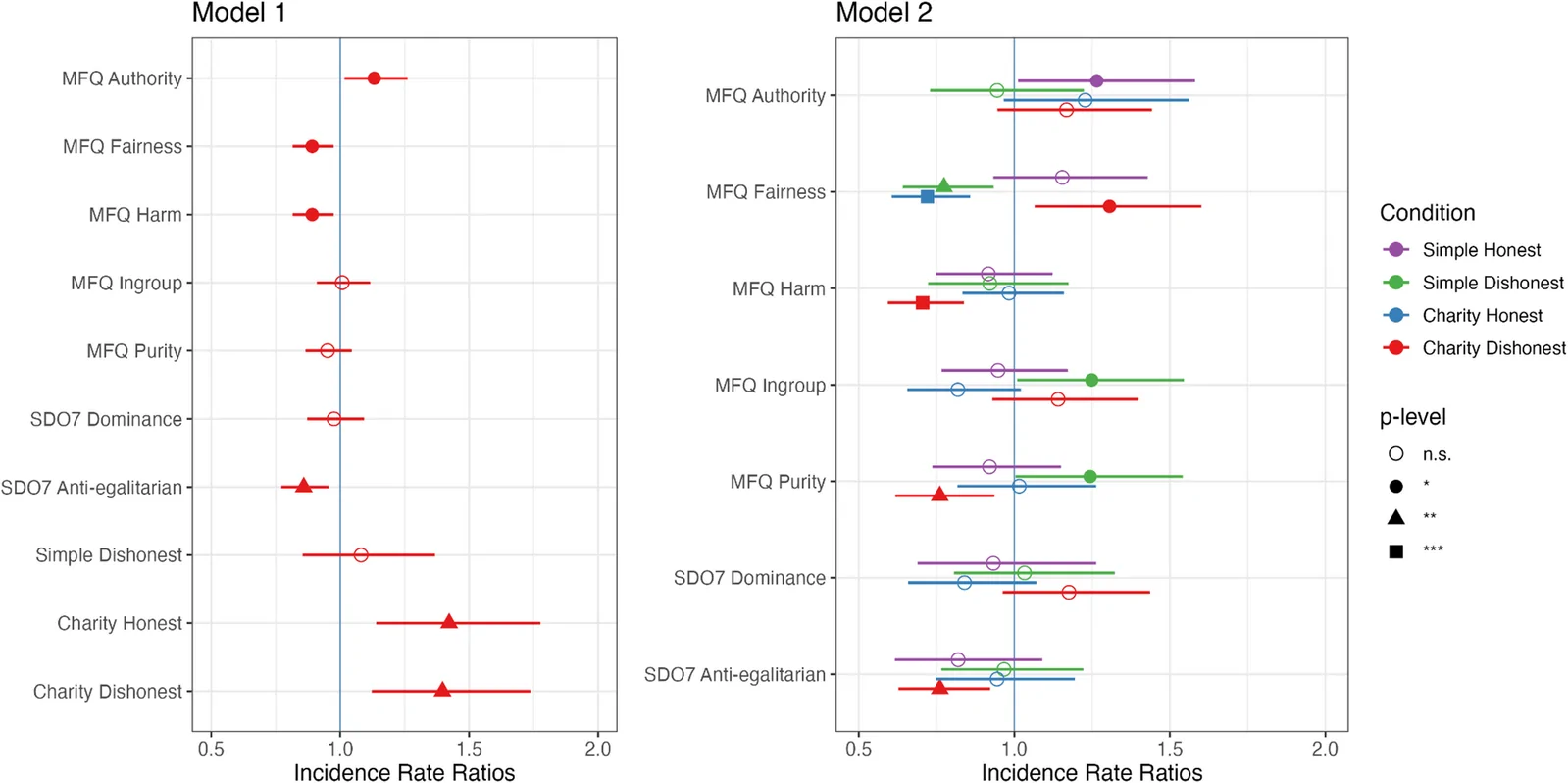
Forest plot of Poisson regression models. Model 1 is a Poisson regression on the effect of psychological traits, and conditions on the number of reported doubles. Model 2 is a series of Poisson regressions on the relationship between the number of reported doubles and psychological traits in the all four conditions. Dots represent the standardised beta values and bars denote the confidence intervals. Separate models were constructed for the four conditions, which are indicated with colours.Significant (*p* < 0.05) correlations are indicated by full dots, *p* < 0.01 indicated by triangles and *p* < 0.001 indicated by squares. The multicollinearity between the variables in both models shows typically low or moderate correlations. The calculated GVIF and VIF values can be found in the Supporting information S9.

To investigate further the effect of psychological traits on cheating behaviour, a series of Poisson regressions was carried out. First, the psychological traits and the condition were set as explanatory variables (Fig. 4 and Supporting information S9, Model 1). In addition to this single model, we investigated four separate models using Poisson regression for each of the four conditions (Fig. 4 and Supporting information S9, Model 2 A-D). Model 1 confirmed previous statistics showing that the participants were more likely to cheat in conditions CH and CD than in condition SH. Furthermore, it showed a positive relationship between MFQ Authority and the number of doubles reported, indicating that people with a strong sense of authority were more likely to cheat. Model 1 also showed a negative relationship between MFQ Fairness, MFQ Harm and SDO7 Anti-egalitarian factors and number of doubles. When Model 2 was used, it turned out that a higher score in MFQ Fairness is associated with a lower level of cheating in SD and CH, and a higher level of cheating in CD; however, no relationship was found in SH. Model 2 revealed further correlations as well: MFQ Harm, MFQ Purity and SDO7 Anti-egalitarian showed a negative relationship with cheating in condition CD, and MFQ Ingroup showed a positive relationship with cheating in condition SD.

## Discussion

We designed a 2 person dice-rolling game, building on Weisel and Shalvi^12^,to examine how the presence of a dishonest partner and opportunities for by-product altruism shape dishonest behaviour. Our first and rather unexpected result was that our participants did not cheat in all four settings, which diverged from the findings of previous similar experiments.

In the case of the settings without charity (i.e. Simple game: SH, SD), the extent of cheating was not significant, that is, the number of doubles did not differ significantly from chance, while in the corresponding setting of the original Weisel and Shalvi experiment^12^ a significant level of cheating was observed. This may be due to the difference in the educational and social background of the participants in our experiment, as has been suggested in another study, too^24^. In the original experiment, the participants were economics students, whereas in ours they were mainly biology and psychology students. For the latter group, maintaining a positive self-image may be more important than maximising financial gain. In contrast, economics students may well be more reward-motivated due to their training^24^. This pattern corresponds to the idea that individuals differ in how they approach lying decisions, ranging along a continuum between two extremes: economic types and ethical types^35,36^. Although the authors find it unlikely, cultural differences may also be behind the lower levels of cheating in our study^37^. Finally, the generally low levels of dishonesty may be due to the relatively small amount of money at stake^25,38^.

Another surprising result in the SH (Simple-Honest) setting is that while the number of doubles was not significantly different from the expected value of truthful reporting, the distribution of reported die rolls was significantly different from the uniform distribution. On closer analysis of the data, it appears that players reported more sixes and fewer ones than would be expected from a uniform distribution (Fig. 3b and Supporting Information, Table S3). This could easily be the result of a sampling fluctuation, but it could also be the case that some players did not understand the game accurately and believed that if they reported a higher number they were more likely to win, regardless of the partner’s roll.

Our principal finding is that dishonest behaviour (cheating) was more prevalent when this behaviour’s by-product was a cost-free altruistic action. This effect is probably due to by-product altruism compensating for negative emotions, such as guilt, that are triggered by unethical behaviour^37^. Further, our results show that the possibility of donating to a charity has a more pronounced effect on dishonest behaviour than observing the actions of a dishonest partner (since the latter did not show a significant effect). A possible explanation for this result is that moral distress and relatively low winnings prevent cheating in settings where cost-free charity is not included. Cost-free charity resolves this moral tension which results in significant levels of cheating. These results imply that reframing collaborative dishonesty as socially beneficial can alleviate the moral distress experienced by dishonest actors. In concordance with this conclusion, a series of studies found that different lies activate different brain regions (e.g. selfish and prosocial, or reputation-risky, detectable and reputation-safe, non-detectable lies)^23,39,40^.

These observations may explain why collaborative dishonesty or corruption occur most in cases when social norms have deteriorated. Examples could be that an act of corruption does not violate the norm extensively, the corrupt act is less likely to be found out, or there is a prosocial element masking the selfish motivation behind dishonest or corrupt behaviour^41^. Consistent with our findings, populist establishments often present their corrupt activities as helping national capitalism and consequently the development of the whole country^42^, transforming an act of selfish corruption into a prosocially motivated act in the mindset of society and consequently making the corrupt regime itself easier to accept. In fact, this argument, though with an opposite cause-effect relationship, follows the same logic as the reasoning that prosocial behaviour is often motivated by selfish goals^43^. Here we say that selfish behaviour is justified by a by-product altruistic benefit. The observation that the dishonest partner had no significant effect on the level of cheating and that we found no evidence of moral erosion could be the consequence of the relatively small amount of money that participants could win, and/or that 20 rounds was not a long enough series to realise that the partner was definitely a cheater (see the bottom row of Fig. 1). The latter possibility is supported by the fact that we found no increase in the number of doubles during sessions with a dishonest partner (Fig. 3a).

Since cheating is a highly moral issue in general, and in this experimental situation too, we expected the moral attitudes of the participants to be related to the expression of dishonest behaviour. Our analysis identified this relationship in a complex and sometimes contradictory way. We expected the Harm domain to be unrelated to the extent of cheating or, if we examine its effect by condition, to be positively correlated with it in the Charity conditions. The analyses showed the opposite: overall, a higher Harm score acts against cheating (Fig. 4, Model 1), which is primarily determined by the strong negative correlation between the CD setting and Harm score (compare Fig. 4 Model 1 with Model 2). This result suggests that those who more strongly reject harm are also more resistant to moral challenges, which is strongest in the CD setting. Further, we expected the *Fairness/reciprocity* domain to be associated with less cheating, as people high in fairness would have a more difficult time violating the rules. Although not entirely clear, our analysis tends to confirm this assumption rather than reject it, as Model 1 and Model 2 showed a correlation in this direction in most cases. We expected the moral domain *Authority/respect* to be associated with less cheating, due to a respect for the rules of the game, but no significant correlations were found in these two cases. Based on our results, the opposite correlation seems more likely, but the relationship is weak, as Model 1 measured a positive correlation, while Model 2 measured a positive relationship only in the SH setting (where there is no significant difference in the number of doubles). We expected that social dominance orientation (SDO) factors would be associated with a higher propensity for cheating. To our surprise, instead of finding such an association, we have found the opposite effect according to Model 1 and Model 2 for the CD setting. It can be concluded that the relations between the measured psychological traits and the extent of cheating is complex and often contradictory. Further targeted research is needed in this area in order to accurately map these relationships.

As in all cases, there were limiting factors in this series of experiments. Most importantly, even the highest possible reward was probably too small to stimulate intensive cheating, although it was roughly the same as in the original WS experiment. Furthermore, because of the COVID pandemics, the subsequent lock-downs and the consequent reluctance of people to get in contact with strangers, the experiment took years to complete. During this extended period, and especially because of the pandemic hitting the economy, inflation further decreased the value of the target yield.

## Conclusion

Our primary result is that by-product altruism had a larger effect on participants’ cheating behaviour than moral erosion caused by the dishonesty of the partner in our experimental setting. Dishonest behaviour is related to measured psychological characteristics in a complex way, but overall, it appears that higher values on the MF subscales and on the SDO7 scale result in a lower propensity for cheating. Putting our results in a broader context, unethical, corrupt, or immoral behaviour may all feel less unacceptable if the parties use their acts benefitting socially prestigious activities or communities as a pretext or excuse for cheating – in addition, of course, to the chance to help themselves selfishly.

## Methods

### Disclosures

We preregistered a full Methods section and analysis scripts on Open Science Framework, together with the materials (code for the game, questionnaires, etc.) or pictures of the materials we used. All materials can be downloaded from: https://osf.io/vsfnd. All experiments were performed in accordance with relevant guidelines and regulations. Informed consent was obtained from all the participants.

The study was approved by the Hungarian United Psychological Research Ethics Committee (Egyesített Pszichológiai Kutatási Etikai Bizottság, Ref. No. 2019/07).

### Participants

Participants were recruited mainly from the Faculty of Science of Eötvös Loránd University, through flyers (see Supporting information S1) and calls for participation in Facebook groups. Most participants were university students, and mostly undergraduates of biology, chemistry, geography and psychology. Recruitment started on September 15, 2019, and ended on November 21, 2023. Our plan was to have the same researcher conduct all experiments. However, because of the COVID-19 pandemic, and the consequent closing of the university building, the experiment took much longer than expected.

The mean age of the participants was 21.3 years (± 3.0 years), 24 women and 12 men for SH, 22.3 years (± 5.3 years), 24 women 11 men and 1 didn’t give her/his gender for SD, 21.9 years (± 4.5 years), 21 women and 15 men for CH and 24.9 years (± 9.1 years), 27 women and 9 men for CD setups. The four settings did not differ in terms of age (ANOVA, *F*(3, 282) = 2.635, *p* = 0.052) and gender ratio (Chi-squared test, ^2^(6,166) = 5.346, *p* = 0.500) either. Participants who did not finish the experiment for any reason or played in a nonconventional way (two participants due to the computer freezing, one participant because he possibly looked into the code, and one because she did not accept the reward for the game) were excluded from data analysis. We kept recruiting participants until we reached the sample size specified by the power analysis for our confirmatory data analysis: 36 participants per condition were randomly assigned to one of the four experimental settings. The only person who communicated with the participants was unaware of which of the four treatments each participant had been assigned to, creating a double blind situation.

For more details about the experimental setting and procedures, see Supporting information S2.

## Experimental procedures

### Overview

The study employed a collaborative dice-rolling game adapted and modified from Weisel and Shalvi^12^ to examine participants’ decision-making behavior under conditions allowing for potential dishonesty. Participants were individually recruited, mostly from universities, and randomly assigned to one of four experimental conditions in a double-blind design. After providing informed consent, participants completed the task alone in a controlled experimental room, interacting only with on-screen instructions. They were told they were paired with another participant online (in reality, the partner’s behaviour was preprogrammed) and played 20 rounds of a collaborative dice-rolling game in which monetary rewards depended on matching the reported outcomes of their partner. One round was randomly chosen for payment at the end of the game. Following the task, participants completed standardised questionnaires assessing psychological traits. For more details, see below and the Supporting information.

### The game

At the beginning of the game participants were told that they get 200 HUF (about 0.5 EUR) for appearing on time, but they would have the opportunity to win extra money by playing a game. After providing their age and gender, participants were assured of anonymity and not being observed, and received instructions on rolling the dice with the cup and peeking through the hole in the lid to read the value.

The rules of the game were explained on the screens. Participants were told that their partner in the game, with whom they would play online, was in another university (in fact, there was no other player, just the computer programme). Following the method presented by Weisel and Shalvi^12^ they were also told that the number of rounds would be more than 10 but less than 30 (in fact, the number of rounds was always exactly 20).

Participants either played the Charity game or the Simple game. To participants who played the Charity game, it was explained that if they won some money at the end of the game, a charity foundation also received a donation of 300 HUF. They were shown three options for local charities to choose from (Rex Dog Shelter Foundation, Together for Children with Leukaemia and the Pearl Foundation for the Abolition of Child Poverty).

Participants in both games were told that they were the second players (Player B) in each round: their partner (Player A) would roll the die first, and report its value which would appear on the screen before their turn to roll the die and enter their value. Both players got a reward if their reported die rolls were of equal value, and none got anything if not. If Player B reported the same number as Player A did, they both got the score of 300 HUF times the value of the reported number; if they reported different numbers, their score was 0.

It was explained to the participants that at the end of the game, the computer would randomly select one round and both participants (and the charity) would be paid the score of this single round in HUF. In other words, a reward was only guaranteed if Player B reported the same values as Player A in every single all of the round.

Participants could test the reward calculations on five practice screens. Finally, participants played 20 rounds of the game. After each round the score appeared on the screen, along with the possible donation sum and beneficiary, if applicable. After 20 rounds the computer randomly selected one round and its score stayed on the screen for the experimenter to see.

The experiment was implemented in z-Tree. The corresponding files can be downloaded from the preregistration. In this we followed Weisel and Shalvi^12^, and most of the screens are translations of the screens they used in the original experiment. Screenshots and the translation of the scripts are provided in Supporting information S6.

### Psychological traits

The Moral Foundations Questionnaire^29,30^(MFQ) and the seven-item Social Dominance Questionnaire^32,34^(SDO7) were recorded. MFQ contains five subscales (Authority underlies virtues of leadership and followership, Fairness underlies the virtues of justice and rights, Harm underlies the virtues of kindness, gentleness, and nurturance, Ingroup underlies the virtues of patriotism and self-sacrifice for the group, and Purity underlies the virtues of self-discipline, self-improvement, naturalness, and spirituality). SDO7 measures the participant’s support for social hierarchy and contains two subscales (The *Dominance* subscale indicates when participants value in-group dominance over out-group, and *Anti-Egalitarianism subscale* indicates valuing nonegalitarian, hierarchical relationships between groups), and the average of the items represents overall social dominance. For more details see the Supporting information S7.

### Statistical analysis

#### Power analysis

In designing the experiments, we used power analysis based on previous papers^12,24^ to estimate the sample size. A detailed description of the analysis can be found in Supporting information S8.

### Data analysis

Our null hypothesis was that the reported values came from a uniform distribution, i.e. numbers from 1 to 6 would be reported with the same probability, since we used an unloaded dice (previously tested, Chi-square goodness-of-fit test, *n* = 703, *p* = 0.9198). We used Chi-square goodness of fit tests to test whether the reported values come from a uniform distribution, separately for each condition. We specified *simulate.p.value = TRUE*, so the *chisq.test* R function used a test statistic and *p* value based on a Monte Carlo approach. In this case, there was no assumed chi-square distribution for the test statistic, so there was no degree of freedom (df) parameter involved.

We tested whether the number of doubles is higher than its expected value with a one-sided one-sample Wilcoxon signed-rank U test in each condition using the *wilcox.test* function of the *stats* R package.

We compare the number of doubles of two conditions with one-sided two-sample Mann-Whitney U tests using the *wilcox.test* function of the *stats* R package.

We compared the number of doubles in the four conditions with a Kruskal-Wallis test using *kruskal.test* function from the *stats* package.

We tested the effect of predictors with Poisson regressions, using the *glm* function from the *stats* R package. The dependent variable was the number of reported doubles (interval) and the predictors were the presence of charity (binary) and partner honesty (binary).

### Exploratory data analysis

We did not preregister data analysis regarding the questionnaire data. To study the relationship between dishonesty and psychological traits, a series linear model was fitted for the full data and for the four conditions separately, where the dependent variable was the number of doubles reported by the participants, and the explanatory variables were the MFQ Authority, MFQ Fairness, MFQ Harm, MFQ Ingroup, MFQ Purity, SDO7 Dominance and SDO7 Egalitarian subscales. To fit the linear model we used the *lm* function from the *stats* R package, and to obtain *p* values we used the *summ* function from the *jtools* R package. The results were reported in the Supporting information S9.

## Supplementary Information

Below is the link to the electronic supplementary material.

Supplementary Material 1

## Data Availability

All our data can be downloaded from here: https://repo.researchdata.hu/privateurl.xhtml? token=53a8049d-4578-4ee5-9eb0-b4e28539c042.
