# Supplementary material for: Cost-free charity facilitates dishonesty in a dice-rolling experiment
Source: Sci Rep. 2026 Jun 19;16:28037. doi: 10.1038/s41598-026-56788-9 (PMC13550399; doi:10.1038/s41598-026-56788-9)
Supplement: Supplementary file 1 [file 41598_2026_56788_MOESM1_ESM.docx]

Supporting information

Cost-free charity facilitates dishonesty in a dice-rolling experiment

Judit Mokos, Anna Fedor, Dorottya Deli, Borbála Kívés, Zsóka Vásárhelyi, Gergely Boza, István Scheuring

## S1. Flyer used for recruiting participants

**Figure S1. Original recruiting flyer in Hungarian - see translation below.**

WE ARE LOOKING FOR PARTICIPANTS!

- 200 HUF if you show up, and possibly 2000 HUF if you are lucky!
- The experiment takes about 45 minutes
- ELTE TTK South Building *[location]*

Contact us at [human.kiserletek@gmail.com](mailto:human.kiserletek@gmail.com) *[human.experiments@gmail.com]*

## S2. Experimental setting and procedures

The experimenter made an appointment with participants in e-mail and met them one-by-one at the Faculty of Plant Systematics, Ecology and Theoretical Biology, where our experimental room was situated. The experimenter followed a written protocol (see Supporting information S3) for conducting the experiments, including the scripts for what to say to the participants.

The experimenter asked the participant to sign the informed consent form (see Supporting information S5) and then to take a random four-digit number from an envelope of number cards that would be used to connect the results of the game with the questionnaires. The participants took their number with them after the experiment. The experimenter then told the participant that all instructions for the game would be on screen and that she would be in the opposite room should the participant have questions (see Supporting information S6). Then she asked them to come and get her when the game was over.

After this, the experimenter left the room and the participant went through the steps of the game alone. When the game was over, the participant informed the experimenter. She went back to the experimental room, where the last screen on the computer showed the reward that the participant won during the game. The experimenter wrote down the reward on the number card.

The experimenter then opened a Google Form in the browser, which contained the Moral Foundations Questionnaire (Graham et al, 2011; Hadarics and Kende, 2017) ( see Supporting information S7) and the Social Dominance Questionnaire (Faragó and Kende, 2017; Ho et al. 2015) (see Supporting information S7). The experimenter left the room until the participant filled out the questionnaires. Finally, the experimenter paid the participant, they signed a receipt and then she concluded the experiment. DD ran the experiments between 8th of October 2019 and 16th February 2021, BK ran the experiments between 4th of October 2021 and 20th of April 2023, JM ran the experiments between 16th of May 2023 and 24th of November 2023. The effect of the experimenter on the game-behaviour were tested and no effect was found (see Supporting information S9).

The experimental room is about 3x4 m, with white walls without any objects and non-necessary furnitures, The experimental desk is to the right of the door with a chair and all other materials necessary for the experiment: a notebook, a mouse, a lidded cup with a hole on top and a dice in it and a sheet of paper with the informed consent. (For pictures of the experimental room and all the equipment used, see Supporting information S4)

## S3. English translation of the protocol

### I. Preparations

*I open everything in the laptop: the questionnaire in the browser and the appropriate version of the game for the next participant. I prepare an informed consent form and a pen. I place the number cards and the dice-rolling set on the desk.*

### II. Greetings and introductions

*I met the participant in front of the entrance of the department.*

Hi, I am Dorka Deli (Borbála Kívés, Judit Mokos), the experimenter. Thanks for coming, please, come in. The experiment will take about 40-45 minutes. Would you like to use the restroom before that?

*We shake hands, then we go into the department. I show them the restroom and then lead them to the experimental room.*

### III. The beginning of the experiment

*I show the participant where to put their coats, bags, etc. The participant receives the informed consent form and a pen.*

Come on in, put your jacket and bag down on this desk. Please take a seat in front of the laptop, and read the informed consent form carefully, and sign it if you agree.

*The participant sits down, reads and signs the informed consent form. Then, I ask them to take a number card and explain what it is for. I show them how to use the dice set and I show them the other room where I will stay during the game.*

Thanks. Please, take a number card from this envelope! You will have to type this number in at the beginning of the game, and also later, into a questionnaire. Since both the game and the questionnaire are anonymous (the only personal data we ask for is your age and gender), we need this number to identify which questionnaire belongs to which game.

You will play a dice game in pairs. You will use this set: shake it, peek in at the top hole, and read the number you rolled. Your partner sits at another university’s experimental room and the two computers are connected.

*I check the time on my mobile and pretend to wait for the partner.*

It seems that your partner is already there, waiting for us to check in. From now on you will find all the instructions on the screen. During the game, I will be in the opposite room, where the door is open. If you have any questions, feel free to pause the game and call me.

*I leave the experimental room and come back only when the participant calls me. I note the starting time in the diary.*

### VI. Questionnaires and finger measurements

*After the participant finished the game, I go back to the experimental room, write down the reward that the participant won on the number card, answer the questions they may have, and then show them the questionnaire.*

Your won x forints, which I write on your number card. You will receive your prize at the end of the experiment, after the questionnaire.

Please follow the instructions on the screen and fill in the questionnaire. There are no right or wrong answers, so please answer the questions honestly and use your best judgement. Responses will be treated anonymously, similar to other data. I leave the room again, please call me when you're done with the questionnaire.

*I leave the room again, until the participant finishes the questionnaire.*

*At the end of the experiment I measure the second and fourth fingers of the participant with the calliper. I type the results into the questionnaire.*

Finally, I will measure your second and fourth fingers on your right hand with this calliper. This is completely painless and takes a maximum of two minutes. Please put your right hand on the table. Your measurements will be treated anonymously just like all the other data.

### We emphasize here that although we measured the ratio of the second and fourth digits in all test subjects and examined whether this ratio correlates with the propensity to cheat in different settings (see below), we omitted these tests from the article. There are three reasons for this. 1) We did not register this experiment in advance. 2) We did not obtain any significant results, and 3) due to space limitations, it was not possible to write about this in more detail.V. Payment, end of experiment

*I pay the participant the amount they won, and ask them to sign the receipt. I thank them for participating and then I show them out of the department.*

Thank you for participating in the experiment! Please do not talk about the details of this experiment with others until the end of the project, February 2020. If you wish, we can give you more information about the goals and the results of the experiment after the project is finished. In this case, please give me your email address.

*If they wish, I write down their email address. I answer any questions they might have, then show them out of the department, and say goodbye.*

### VI. Finishing

*I write down the finishing time into the diary, along with the participant’s questions and anything unusual that might have happened during the experiment. I save the results from the ztree folder to the Results folder. I prepare the materials for the next participant.*

## S4. Pictures of the experimental room and materials

**Figure S2. The experimental room and the desk with all the materials: the computer, the informed consent form, the dice rolling cup and the digital calliper.**

**Figure S3. The dice rolling cup with a hole on top, and the digital calliper used for digit ratio measurements.**

## S5. English translation of the informed consent form

INFORMATION ABOUT THE EXPERIMENT

Within the research project NKFI K128289 we are conducting experiments to study certain aspects of human behaviour. During the experiment, you will play a dice game with another participant at another university through the internet. After the game, you will be asked to fill out a short questionnaire, then we will measure your index finger and ring finger.

For showing up, you will get 200 HUF. You can win more money during the game, which we will pay you right after the experiment. The experiment takes about 45 minutes.

Participating in this experiment is voluntary and anonymous. We will ask your age and gender, but no personal data that could be used for identification. Your data will be published as part of a bigger database and will be statistically analysed. We will publish the results of this study in scientific journals and in other media. Our experimental protocol was approved by the United Psychological Research Ethics Committee. Dr István Scheuring is responsible for the data control. If you have any problems, you have a right to complain about the handling of your personal data at the [strategia@rk.elte.hu](mailto:strategia@rk.elte.hu) email address.

We will not reveal the goal of this experiment here or during the experiment. This is necessary for the study to yield reliable results. You can get fully informed about the experiment at the end of this study if you write to [human.kiserletek@gmail.com](mailto:human.kiserletek@gmail.com).

If you feel uncomfortable during the experiment, you can quit at any point without consequences.

If you have further questions or comments regarding the experiment, please, contact the principal investigator Dr. István Scheuring (Evolutionary Systems Research Group, Centre for Ecological Research & MTA-ELTE Theoretical Biology and Evolutionary Ecology Research Group) at [istvanscheuring@gmail.com](mailto:istvanscheuring@gmail.com).

CONSENT FORM

I have read the information about the experiment.

Yes / No

By agreeing to participate in this study I consent to my data being used anonymously, as part of a database, in this research project.

Yes / No

I am aware that I can quit the experiment at any time.

Yes / No

Date:.......................................................

Name:.....................................................

Signature:...............................................

## S6. The game

*Below, we provide the English translation of the screens of the game, and some screenshots of the actual screens (with the original Hungarian instructions).*

### Screen 1

Welcome and thank you for participating in the experiment!

Please, turn off or mute your mobile phone!

Read the instructions carefully. The experimenter will wait in the opposite room while you finish the following game. If you have any questions, open the door and call the experimenter!

For showing up, you will get 200 HUF. If any time you feel that you want to finish the experiment, feel free to call the experimenter. In this case you will still get the 200 HUF.

During the experiment you can win further money.

### Screen 2

Please, type here the 4 digit number you have drawn before the experiment.

When you have finished, press FORWARD!

### Screen 3

Please, mark your gender and type your age. We will record no other personal data during the experiment. The experiment is **not** recorded on video, and no one is observing you in any way.

If you are ready for the experiment, press FORWARD!

### Screen 4

**Game instructions**

During the game your reward depends on luck, determined by dice rolls. You will have to roll a dice, and type the result of the roll into the computer. The reward of each roll will be determined by the rules shown on the screen.

**How to roll?**

On the desk you find a cup with a hole on the top, with a six sided dice in it. Take the cup, shake it, and put it back on the desk. Then peek through the hole to see which number you rolled. During the game this number is the one you will have to type into the computer.

Make sure the dice is regular and try rolling a couple of times!

### Screen 5 (charity condition)

**The course of the game**

You are playing with somebody else, who is in another university. The reward depends on both of your and your partner’s roll. If you roll the same number, you both get a reward, otherwise neither of you get a reward. Your partner will always be the first to roll, and you will be the second. Before you roll, we will show you on the screen what number your partner rolled. We will also show you the table containing the rules determining the rewards. You will play several rounds with the same partner. You will play at least 10 rounds, but not more than 30. In each round we determine the rewards separately.

**At the end of the game the computer will randomly choose one round you played, and you and your partner will get the reward of that round.**

If at the end of the game the computer draws a round with rewards, a charity foundation, which you can choose from our list, gets a donation of 300 HUF. In this case your partner also chooses a charity foundation, which also gets a donation of 300 HUF. At the end of the project we will transfer the sum of the final rewards to the chosen foundations.

Press FORWARD and see the list of foundations you can choose from.

### Screen 6 (charity condition)

The supportable foundations are as follows:

**Rex Dog Shelter Foundation**

The foundation’s goals are providing harmonious living between humans and animals, educating responsible animal keeping and preventing the suffering of animals.

**Together for the Children with Leukaemia**

The foundation’s goal is to improve the circumstances of children in Hungary living with leukaemia or other type of cancer, and to enhance their chances of recovering.

**Real Pearl Foundation**

An organisation working for equal opportunities, social integration, and overcoming child poverty and inherited extreme poverty.

### Screen 7

**The reward**

The table shows the rules determining the reward. The table contains the first roll (your partner’s roll), the second roll (your roll) and the associated reward.

Most importantly, the players can only get a reward, if the two rolls are identical. If the two rolls differ, they get nothing. If the two rolls are identical, they get as many times 300 HUF, as the value of the roll (e.g. if both rolled a 2, each of them get 600 HUF).

On the next page you will get a chance to practise the rule.

*[TABLE 1^st^ row: Your partner's roll, Your roll, Your partner’s reward, Your reward]*

**Figure S4. Screen 7. The table contains the reward rules.**

### Screen 8

**Practice rolls (not giving reward)**

In the following, during five rounds, you can practise the game. This is the first round. Please roll for both players and type in the numbers! Then press FORWARD to see the reward. The practice rounds don’t give real rewards.

You can also type numbers without rolling.

Your partner’s roll:

Your roll:

**Figure S5. Screen 8. Instructions for practice rounds and the reward table.**

### Screen 9 (charity condition)

**Practice rolls (not giving reward)**

You can practise four more times. For the next practice game press FORWARD.

Your partner’s roll: 2

Your roll: 2

Your reward: 600

In this round you **both rolled a 2**, thus this round would mean **600 HUF reward** each. The foundation would get 300 HUF donation.

**Figure S6. Screen 9. Rewards from the previous practice round and the reward table.**

*[3 more practice rounds with similar layouts]*

### Screen 10 (charity condition)

**Practice rolls (not giving reward)**

You can practise one more time. For the next practice game press FORWARD.

Your partner’s roll: 2

Your roll: 1

Your reward: 0

In this round you rolled **differing** numbers, thus this round would mean **0 HUF reward** each. The foundation would not get any donations.

### Screen 11

**Beginning of the game**

Now you will begin the rolls that determine real rewards. Please, follow the instructions on the screens accurately!

Wait until your partner is also ready for the game! This can take a while.

Don’t forget, at the end of the game the computer will randomly choose one round you played, and you and your partner will get the reward of that round.

### Screen 12

**Beginning of the game**

Now you will begin the rolls that determine real rewards. Please, follow the instructions on the screens accurately!

Your partner is ready for the game. If you are also ready, press BEGIN!

Don’t forget, at the end of the game the computer will randomly choose one round you played, and you and your partner will get the reward of that round.

*[We show the supportable foundations again, and on the next screen participants choose which they want to support if they get a final reward.]*

### Screen 13

**Roll for reward**

Wait until your partner has rolled!

### Screen 14

**Roll for reward**

Your partner has typed in his result. Please, now roll exactly once, type in the result and press FORWARD!

Your partner’s roll: 6

Your roll:

*[In case of typing 8, error message: The largest number you can enter into the “Your roll” area is 6.]*

### Screen 15 (charity condition)

**Roll for reward**

Your reward: 1800

Your partner’s roll: 6

Your roll: 6

In this round you **both rolled a 6**, thus this round means **1800 HUF reward** each. The foundations would get 300 HUF donations each.

The game continues. For starting the next round, press FORWARD!

### Screen 16 (charity condition)

**Roll for reward**

Your reward: 0

Your partner’s roll: 6

Your roll: 1

In this round you rolled **differing** numbers, thus this round means **0 HUF reward** each. The foundations do not get any donations.

The game continues. For starting the next round, press FORWARD!

### Screen 17

**The game is over**

The computer now randomly chooses a round, the reward of which you and your partner get. For the result of this draw press FORWARD.

Below you can see the results of the rounds you played.

**Figure S7. Screen 17 A table showing all the played rounds with the corresponding rewards.**

### Screen 18 (charity condition)

**The game is over**

The computer has randomly chosen a round, the reward of which you will get. Thank you for participating in this experiment! The game is over. Next, we will ask you to fill out a questionnaire. Please, call the experimenter, who will note your reward and open the questionnaire for you.

The randomly chosen round: 5

Your final reward: 1800

In the randomly chosen round you **both rolled a 6**, thus you won **1800 HUF each**. The chosen foundations got 300 HUF donations each.

## S7. Questionnaires

### Moral foundations questionnaires

*We have used the Moral Foundations Questionnaire (*Graham et al. 2011*) translated to Hungarian by Hadarics & Kende (2017). Note, that in the Hungarian version of the questionnaire the two parts come in reverse order compared to the English version. Below is the questionnaire in English, in the order we used it.*

Part 1. Please read the following sentences and indicate your agreement or disagreement:

[0]	[1]	[2]	[3]	[4]	[5]

Strongly	Moderately	Slightly	Slightly	Moderately	Strongly

disagree	disagree	disagree	agree	agree	agree

______1. Compassion for those who are suffering is the most crucial virtue. (harm)

______2. When the government makes laws, the number one principle should be ensuring that everyone is treated fairly. (fairness)

______3. I am proud of my country’s history. (ingroup)

______4. Respect for authority is something all children need to learn. (authority)

______5. People should not do things that are disgusting, even if no one is harmed. (purity)

______6. It is better to do good than to do bad. (catch)

______7. One of the worst things a person could do is hurt a defenceless animal. (harm)

______8. Justice is the most important requirement for a society. (fairness)

______9. People should be loyal to their family members, even when they have done something wrong. (ingroup)

______10. Men and women each have different roles to play in society. (authority)

______11. I would call some acts wrong on the grounds that they are unnatural. (purity)

______12. It can never be right to kill a human being. (harm)

______ 13. I think it’s morally wrong that rich children inherit a lot of money while poor children inherit nothing. (fairness)

______ 14. It is more important to be a team player than to express oneself. (ingroup)

______ 15. If I were a soldier and disagreed with my commanding officer’s orders, I would obey anyway because that is my duty. (authority)

______ 16. Chastity is an important and valuable virtue. (purity)

Part 2. When you decide whether something is right or wrong, to what extent are the following considerations relevant to your thinking? Please rate each statement using this scale:

[0] = not at all relevant (This consideration has nothing to do with my judgments of right and wrong)

[1] = not very relevant

[2] = slightly relevant

[3] = somewhat relevant

[4] = very relevant

[5] = extremely relevant (This is one of the most important factors when I judge right and wrong)

______17. Whether or not someone suffered emotionally (harm)

______18. Whether or not some people were treated differently than others (fairness)

______19. Whether or not someone’s action showed love for his or her country (ingroup)

______20. Whether or not someone showed a lack of respect for authority (authority)

______21. Whether or not someone violated standards of purity and decency (purity)

______22. Whether or not someone was good at math (catch)

______23. Whether or not someone cared for someone weak or vulnerable (harm)

______24. Whether or not someone acted unfairly (fairness)

______25. Whether or not someone did something to betray his or her group (ingroup)

______26. Whether or not someone conformed to the traditions of society (authority)

______27. Whether or not someone did something disgusting (purity)

______28. Whether or not someone was cruel (harm)

______29. Whether or not someone was denied his or her rights (fairness)

______30. Whether or not someone showed a lack of loyalty (ingroup)

______31. Whether or not an action caused chaos or disorder (authority)

______32. Whether or not someone acted in a way that God would approve of (purity)

### Social dominance orientation questionnaire

*We have used the Social Dominance Orientation questionnaire (Ho et al. 2015) translated to Hungarian by Faragó & Kende (2017). Note, that the Hungarian version lists the statements in a different order than the English version. Below is the questionnaire in English, in the order we used it.*

Show how much you favour or oppose each idea below by selecting a number from 1 to 7 on the scale below. You can work quickly; your first feeling is generally best.

**1** = Strongly Oppose

**2** = Somewhat Oppose

**3** = Slightly Oppose

**4** = Neutral

**5** = Slightly Favour

**6** = Somewhat Favour

**7** = Strongly Favour

______1. An ideal society requires some groups to be on top and others to be on the bottom. (dominance)

______2. Some groups of people must be kept in their place. (dominance)

______3. It’s probably a good thing that certain groups are at the top and other groups are at the bottom. (dominance)

______4. Some groups of people are simply inferior to other groups. (dominance)

______5. Groups at the bottom are just as deserving as groups at the top. (reversed) (dominance)

______6. No one group should dominate in society. (reversed) (dominance)

______7. Groups at the bottom should not have to stay in their place. (reversed) (dominance)

______8. Group dominance is a poor principle. (reversed) (dominance)

______9. We should not push for group equality. (antiegalitarian)

______10. We shouldn’t try to guarantee that every group has the same quality of life. (antiegalitarian)

______11. It is unjust to try to make groups equal.(antiegalitarian)

______12. Group equality should not be our primary goal. (antiegalitarian)

______13. We should work to give all groups an equal chance to succeed. (reversed) (antiegalitarian)

______14. We should do what we can to equalise conditions for different groups. (reversed) (antiegalitarian)

_______15. No matter how much effort it takes, we ought to strive to ensure that all groups have the same chance in life. (reversed) (antiegalitarian)

_______16. Group equality should be our ideal. (reversed) (antiegalitarian)

## S8. Power analysis

We used Monte Carlo simulations to estimate the sample sizes (see MonteCarlo.Rmd). Our goal was to achieve at least 90% power on all of the planned statistical tests that tested our main hypotheses: one-sample and two-sample Wilcoxon signed-rank U tests (main effects; the latter are also called Mann-Whitney U tests). We simulated participant behaviour based on our assumptions and tested the outcome with a series of sample sizes. We ran 10 000 simulations with each sample size and calculated power for a given sample size and a given comparison as the percentage of simulations where the Wilcoxon signed rank U test was significant (*p* < 0.05), i.e., detected a true difference. We decided to choose the smallest sample size with which we could achieve the desired power on all of our tests. The random seed for the simulations was set to be the then current date and time: 2019-10-01 20:13:36.

### Simulating player A behaviour

We planned to test our participants in four conditions with the sequential dyadic dice-rolling task previously used by Weisel & Shalvi (2015). The main difference is that in our experiment, player A is simulated by the computer, although player B is led to believe that he is playing with a real person. Player A is either “honest” or “dishonest”. The values for honest player As will be sampled from a uniform distribution between 1 and 6 (sampling will be done for each player, independently). The reported values for dishonest player A-s will be sampled from the values reported by participants who were in the role of player A in Wouda et al. (2017) in their Study 2: High behavioural norm group. We chose this experiment to be the basis for the simulations because it was preregistered and we managed to communicate with the authors via email.

The original authors sent us their data and script with which they calculated their statistics. In their experiment, participants’ norm was manipulated by showing them results from previous experiments: either from an experiment where participants cheated quite often (High behavioural norm group) or from an experiment where participants cheated less often (Low behavioural norm group). The manipulation affected participant behaviour: Participants in the high behavioural norm group reported higher values more often and reported double rolls more often than participants in the low behavioural norm group.

The distribution of values reported by player As in the high behavioural norm group is shown in Table S1. We used this distribution to sample values for our simulated dishonest player A:

| Value | Frequency |
| --- | --- |
| 1 | 36 |
| 2 | 25 |
| 3 | 41 |
| 4 | 51 |
| 5 | 66 |
| 6 | 181 |

Table S1. The distribution of values reported by player As in the high behavioural norm group

### Simulating player B behaviour

For the estimation of power we simulated the behaviour of player Bs as well. For this, we had to guess what values player Bs would report in our control group and three experimental manipulation groups. Player Bs can only increase their payoff by reporting doubles: if they cheated, they would report more doubles than expected by chance. Apart from doubles, there is no point for player Bs to report higher values than what they actually rolled, except, if they intend to signal to player As, trying to convince them to cheat. We decided not to model this behaviour for the purposes of power calculations, because we don’t know how often this would happen.

We simulated player B behaviour in the following way. For each of our participant groups, we estimated the probability with which player Bs would report doubles. Then we randomly assigned each roll as double or non-double based on this probability. If a roll was a double, player B’s reported value was the same as player A’s value; if a roll was not a double, player B’s reported value was sampled from 1 to 6 with a uniform distribution, excluding player A’s value. For estimating the probability of reporting doubles, we used data from Wouda et al. (2017), Study 2, again. We chose this experiment because their manipulation presumably affected participant behaviour in a similar way as our intended manipulations would do.

#### Simple game with honest partner (control group)

For our simple game with honest partner the probability of reporting a double was taken from player Bs in the Low behavioural norm group in Wouda et al. (2017), Study 2: it was 0.4666667 (see their Table 1). This could potentially overestimate the probability of reporting doubles in our experiment. Differences that might lead to overestimation:

- Participants in Wouda et al. (2017), Study 2 were used to participating in economic studies, which supposedly increases the tendency to cheat. Most of our participants probably did not participate in any behavioural experiments before.
- Despite the norm manipulation, their player As still cheated by reporting higher values more often than expected by chance, which in turn might have influenced player B behaviour to cheat too. Our simulated player As will be perfectly “honest”, which might discourage cheating for player Bs too.
- The norm manipulation probably affected player B behaviour in its own right. Although it decreased cheating compared to the high behavioural norm group, it might have increased cheating compared to no manipulation at all (which was not tested). The figures that participants studied before the experiment showed data from their Study 1, where participants reported 30% of doubles, which is still higher than expected by chance.

Overestimating the probability of reporting doubles might lead to overestimating power for a given sample size with the one sample Wilcoxon signed rank U test - however, we did not worry about this, since the sample size necessary for two-sample Wilcoxon tests will be much higher, i.e., the one sample tests won’t be the ones to define our sample size anyway.

#### Charity game with dishonest partner (double manipulation group)

In our view, player B behaviour in Wouda et al.’s Study 2 was affected in two ways: 1) By the experimental manipulation of showing them figures about the results of previous experiments, and 2) by the behaviour (reported values) of player A, which was also affected by the experimental manipulation. In other words, player B in the high behavioural norm group was “encouraged” to cheat not only by the experimental manipulation but also by the behaviour of player A.

In our charity game with dishonest partner the case is similar: dishonest player A cheats more than honest player A and, also, there is the effect of the experimental manipulation of donating to a charity. The difference between honest and dishonest player A behaviour in our case is higher than the difference between player A behaviour in the high and low behavioural norm group in the Wouda et al. experiment, which could lead to underestimation of the effect, what is a safe thing to do when estimating power. As for the experimental manipulation, we have no way to guess how much charity would affect behaviour in this experiment, so we just suppose that the effect would be similar to that of the norm manipulation in Wouda et al.’s experiment.

For these reasons we took the probability of reporting doubles from player Bs in the High behavioural norm group in Study 2: 0.6275. (The value of 67% reported in Wouda et al., 2017, Table 2 must be a typo.)

#### Single manipulation groups

If we assume that the effect of a dishonest partner and the effect of charity increase cheating additively, then the probability of reporting doubles in our single manipulation groups (simple game with dishonest partner and charity game with honest partner) should be between that of the control group (simple game with honest partner) and the double manipulation group (charity game with dishonest partner). For our power simulations we assumed that the effects of the manipulations are the same: both manipulations are responsible for 50% of the difference between the control group and the double manipulation group.

We note that equal effects yield the highest power on all two-sample tests and might potentially lead to underestimation of sample size. If it turns out that one of the manipulations, e.g., partner honesty, has a stronger effect, then we would have underestimated power for the SH-SD and CH-CD comparisons and overestimated power for the SH-CH and SD-CD comparisons. As a consequence, effect size would be too small for the latter to reach the desired power.

#### Summary

A completely honest player B would report doubles with the probability of 0.1666667. In our power simulations we estimated that player Bs would report doubles in the different experimental groups with the following probabilities:

- Simple game with honest partner: 0.4666667
- Simple game with dishonest partner: 0.5470833
- Charity game with honest partner: 0.5470833
- Charity game with dishonest partner: 0.6275

### Results of the power analysis

After simulating the reported values of participants in this way, we calculated the statistical tests that we planned for our experiments and then calculated power for each comparison as the percentage of tests that detected a true difference (i.e., *p* < 0.05). The results of our simulations are shown in Table S2. The table shows the power of the one-sample Wilcoxon tests (columns 2-5) and the two-sample Wilcoxon tests (columns 6-9). We chose the smallest sample size with which all tests had a power of at least 90%: 36 participants in each group.

Table S2. Simulated results. Power of the one-sample Wilcoxon tests (columns 2-5) and the two-sample Wilcoxon tests (columns 6-9).

| Sample size | SH | SD | CH | CD | SH_SD | CH_CD | SH_CH | SD_CD |
| --- | --- | --- | --- | --- | --- | --- | --- | --- |
| 30 | 100 | 100 | 100 | 100 | 85.32 | 86.01 | 85.22 | 86.52 |
| 31 | 100 | 100 | 100 | 100 | 86.27 | 87.28 | 85.72 | 87.42 |
| 32 | 100 | 100 | 100 | 100 | 87.00 | 88.29 | 86.96 | 87.64 |
| 33 | 100 | 100 | 100 | 100 | 88.19 | 89.22 | 88.14 | 89.25 |
| 34 | 100 | 100 | 100 | 100 | 89.20 | 89.96 | 88.91 | 89.50 |
| 35 | 100 | 100 | 100 | 100 | 89.90 | 90.95 | 90.13 | 90.66 |
| **36** | **100** | **100** | **100** | **100** | **90.42** | **91.37** | **91.10** | **91.95** |
| 37 | 100 | 100 | 100 | 100 | 91.46 | 91.94 | 90.99 | 92.34 |
| 38 | 100 | 100 | 100 | 100 | 92.54 | 91.82 | 91.72 | 91.99 |
| 39 | 100 | 100 | 100 | 100 | 92.88 | 93.18 | 92.75 | 93.12 |
| 40 | 100 | 100 | 100 | 100 | 93.11 | 94.25 | 93.26 | 93.88 |

### Exclusion criteria

Participants who did not finish the game will be excluded from the analysis. We also checked if all the values by both players are between 1 and 6 (other values are only possible if there are bugs in the code; we will exclude all participants if this happens). Participants will not be excluded for other reasons for our confirmatory data analyses. We used our preregistered R markdown script for confirmatory data analysis (stat.Rmd).

## S9. Results

Table S3. The frequencies of computer-generated (Player A) and reported-values (Player B) in the different settings. A series of Chi-square tests were performed to see if the reported values of the participants (Player B) come from a uniform distribution.

|  | Simple Honest | | Simple Dishonest | | Charity Honest | | Charity Dishonest | |
| --- | --- | --- | --- | --- | --- | --- | --- | --- |
| Reported value | Player A | Player B | Player A | Player B | Player A | Player B | Player A | Player B |
| 1 | 117 | 91 | 63 | 112 | 112 | 128 | 56 | 107 |
| 2 | 122 | 117 | 43 | 107 | 132 | 130 | 41 | 111 |
| 3 | 122 | 110 | 82 | 117 | 122 | 114 | 80 | 102 |
| 4 | 112 | 124 | 76 | 135 | 114 | 115 | 90 | 131 |
| 5 | 126 | 133 | 119 | 104 | 117 | 108 | 107 | 117 |
| 6 | 121 | 145 | 337 | 145 | 123 | 123 | 346 | 152 |
| Chi-squared test | χ^2^=14.67, p = 0.012 | | χ ^2^=11.23, *p* = 0.036 | | χ ^2^=3.28, *p* = 0.674 | | χ ^2^=14.4, *p* = 0.011 | |

Table S4. Poisson regressions of the effect of the round on the number of reported doubles.

| **Condition** |  | **Estimate** | **Std. Error** | ***t* value** | ***p* value** |
| --- | --- | --- | --- | --- | --- |
| Simple Honest | (Intercept) | 7.116 | 1.013 | 7.023 | <0.001 |
|  | Round | 0.013 | 0.085 | 0.151 | 0.882 |
| Simple Dishonest | (Intercept) | 7.563 | 1.076 | 7.032 | <0.001 |
|  | Round | 0.008 | 0.090 | 0.092 | 0.928 |
| Charity Honest | (Intercept) | 11.047 | 1.007 | 10.972 | <0.001 |
|  | Round | -0.147 | 0.084 | -1.753 | 0.097 |
| Charity Dishonest | (Intercept) | 9.963 | 1.136 | 8.770 | <0.001 |
|  | Round | 0.037 | 0.095 | 0.388 | 0.702 |

### Corruption and psychological traits

#### Spearman correlations

Table S5 A-D. The correlation matrix of the psychological traits and the number of reported doubles in the four treatments. To calculate the adjusted P values a Benjamini-Hochberg correction was used.

Table S5 A. SD condition

| **Variable** | ***r*** | ***p*** | **adjusted *p*** |
| --- | --- | --- | --- |
| MFQ authority | 0.248 | 0.145 | 0.441 |
| MFQ fairness | -0.334 | 0.046* | 0.260 |
| MFQ harm | -0.125 | 0.467 | 0.757 |
| MFQ ingroup | 0.246 | 0.149 | 0.441 |
| MFQ purity | 0.251 | 0.14 | 0.441 |
| SDO7 dominance | 0.236 | 0.165 | 0.441 |
| SDO7 antiegalitarian | 0.182 | 0.287 | 0.574 |
| SDO7 | 0.244 | 0.151 | 0.441 |

Table S5 B.SH condition

| **Variable** | ***r*** | ***p*** | **adjusted *p*** |
| --- | --- | --- | --- |
| MFQ authority | 0.123 | 0.473 | 0.757 |
| MFQ fairness | 0.378 | 0.023* | 0.247 |
| MFQ harm | 0.021 | 0.902 | 0.964 |
| MFQ ingroup | -0.113 | 0.51 | 0.777 |
| MFQ purity | -0.226 | 0.186 | 0.457 |
| SDO7 dominance | -0.281 | 0.096. | 0.440 |
| SDO7 antiegalitarian | -0.443 | 0.007** | 0.216 |
| SDO7 | -0.383 | 0.021* | 0.247 |

| Table S5 C.CD condition | | |  | | | |  | |  | | |
| --- | --- | --- | --- | --- | --- | --- | --- | --- | --- | --- | --- |
| **Variable** | | ***r*** | | | | ***p*** | | **adjusted *p*** | |  |  |
| MFQ authority | | 0.037 | | | 0.828 | | | | 0.964 |  |  |
| MFQ fairness | | -0.033 | | | 0.848 | | | | 0.964 |  |  |
| MFQ harm | | -0.344 | | | 0.04* | | | | 0.260 |  |  |
| MFQ ingroup | | -0.007 | | | 0.965 | | | | 0.965 |  |  |
| MFQ purity | | -0.204 | | | 0.232 | | | | 0.530 |  |  |
| SDO7 dominance | | 0.041 | | | 0.812 | | | | 0.964 |  |  |
| SDO7 antiegalitarian | | -0.191 | | | 0.264 | | | | 0.563 |  |  |
| SDO7 | | -0.069 | | | 0.69 | | | | 0.964 |  |  |
| Table S5 D.CH condition | | | |  | | |  | |  | | |
| **Variable** | ***r*** | | | | | | ***p*** | | **adjusted *p*** | |  |
| MFQ authority | -0.036 | | | | | | 0.834 | | 0.964 | |  |
| MFQ fairness | -0.331 | | | | | | 0.049* | | 0.260 | |  |
| MFQ harm | -0.138 | | | | | | 0.421 | | 0.748 | |  |
| MFQ ingroup | -0.173 | | | | | | 0.313 | | 0.589 | |  |
| MFQ purity | -0.014 | | | | | | 0.934 | | 0.964 | |  |
| SDO7 dominance | -0.065 | | | | | | 0.706 | | 0.964 | |  |
| SDO7 antiegalitarian | -0.017 | | | | | | 0.922 | | 0.964 | |  |
| SDO7 | -0.047 | | | | | | 0.788 | | 0.964 | |  |
|  |  | | | | | |  | |  | | |

#### Poisson regression, Model 1

A Poisson regression was carried out to study the effect of psychological traits and the conditions on the cheating behaviour. The dependent variable was the number of reported doubles. The explanatory variables were the psychological traits measured by the MFQ and SDO7 questionnaires’ subscales and the conditions (SH vs SD, CH, CD).

To test for multicollinearity, the adjusted generalised variance-inflation factors (GVIF) were calculated, using the *vif* function from the car R package. An adjusted GVIF around 1 indicates no correlation with the other predictors, an adjusted GVIF under 2.25 indicates a moderate multicollinearity, while an adjusted GVIF higher than 5 indicates high multicollinearity.

Table S6. Poisson regression on the effect of psychological traits on the number of reported doubles. *n*=144, χ²(10) = 43.99, *p* = 0.00, *Pseudo-R*² (Cragg-Uhler) = 0.26, *Pseudo-R*² (McFadden) = 0.06 *AIC* = 751.49, *BIC* = 784.16

| **Variables** | **Est.** | **S.E.** | ***z* val.** | ***p*** | **adjusted *GVIF*** |
| --- | --- | --- | --- | --- | --- |
| (Intercept) | 3.38 | 0.51 | 6.65 | 0.00 |  |
| MFQ Authority | 0.17 | 0.07 | 2.26 | **0.02** | 1.446 |
| MFQ Fairness | -0.21 | 0.08 | -2.53 | **0.01** | 1.216 |
| MFQ Harm | -0.20 | 0.08 | -2.52 | **0.01** | 1.215 |
| MFQ Ingroup | 0.01 | 0.07 | 0.15 | 0.88 | 1.349 |
| MFQ Purity | -0.05 | 0.05 | -1.04 | 0.30 | 1.291 |
| SDO7 Dominance | -0.03 | 0.06 | -0.42 | 0.67 | 1.489 |
| SDO7 Anti-Egalitarian | -0.15 | 0.05 | -2.79 | **0.01** | 1.411 |
| SD | 0.08 | 0.12 | 0.65 | 0.52 | 1.045 |
| CH | 0.35 | 0.11 | 3.11 | **0.00** |  |
| CD | 0.33 | 0.11 | 3.00 | **0.00** |  |

#### Poisson regressions, Models 2 A, B, C, D

Four Possion regression were carried out separately for the four treatments. The dependent variable was the number of reported doubles. The explanatory variables were the psychological traits measured by the MFQ and SDO7 questionnaires’ subscales. To test for multicollinearity, the variance-inflation factors were calculated, using the *vif* function from the *car* R package. A *VIF* around 1 indicates no correlation with the other predictors, a *VIF* under 5 indicates a moderate multicollinearity, while a *VIF* higher than 10 indicates high multicollinearity.

##### Simple Honest

Table S7. Poisson regression on the effect of psychological traits on the number of reported doubles. *n*=36, χ²(7) = 20.23, p = 0.01, *Pseudo-R*² (Cragg-Uhler) = 0.43, *Pseudo-R*² (McFadden) = 0.12, *AIC* = 161.65, *BIC* = 174.32

| **Variables** | **Est.** | **S.E.** | ***z* val.** | ***p*** | ***VIF*** |
| --- | --- | --- | --- | --- | --- |
| (Intercept) | 0.80 | 1.43 | 0.56 | 0.57 |  |
| MFQ Authority | 0.36 | 0.18 | 2.07 | **0.04*** | 2.01 |
| MFQ Fairness | 0.33 | 0.25 | 1.32 | 0.19 | 1.72 |
| MFQ Harm | -0.16 | 0.19 | -0.84 | 0.40 | 1.41 |
| MFQ Ingroup | -0.09 | 0.17 | -0.50 | 0.62 | 1.59 |
| MFQ Purity | -0.09 | 0.13 | -0.73 | 0.46 | 2.20 |
| SDO7 Dominance | -0.07 | 0.17 | -0.45 | 0.65 | 3.51 |
| SDO7 Anti-Egalitarian | -0.22 | 0.16 | -1.37 | 0.17 | 3.21 |

##### Simple Dishonest

Table S8. Poisson regression on the effect of psychological traits on the number of reported doubles. *n*=36, *χ*²(7) = 22.99, *p* = 0.00, *Pseudo-R*² (Cragg-Uhler) = 0.48, *Pseudo-R*² (McFadden) = 0.13, *AIC* = 174.81, *BIC* = 187.48

| **Variables** | **Est.** | **S.E.** | ***z* val.** | ***p*** | ***VIF*** |
| --- | --- | --- | --- | --- | --- |
| (Intercept) | 2.10 | 1.18 | 1.78 | 0.08 |  |
| MFQ Authority | -0.07 | 0.17 | -0.43 | 0.67 | 2.40 |
| MFQ Fairness | -0.38 | 0.14 | -2.68 | **0.01**** | 1.62 |
| MFQ Harm | -0.12 | 0.19 | -0.66 | 0.51 | 2.03 |
| MFQ Ingroup | 0.28 | 0.14 | 2.04 | **0.04*** | 1.63 |
| MFQ Purity | 0.24 | 0.12 | 1.99 | 0.05 | 1.37 |
| SDO7 Dominance | 0.03 | 0.14 | 0.25 | 0.80 | 2.23 |
| SDO7 Anti-Egalitarian | -0.03 | 0.11 | -0.28 | 0.78 | 2.26 |

##### Charity Honest

Table S9. Poisson regression on the effect of psychological traits on the number of reported doubles. n=36, *χ*²(7) = 22.04, *p* = 0.00 Pseudo-R² (Cragg-Uhler) = 0.46 Pseudo-R² (McFadden) = 0.10 *AIC* = 207.63, *BIC* = 220.30

| **Variables** | **Est.** | **S.E.** | ***z* val.** | ***p*** | ***VIF*** |
| --- | --- | --- | --- | --- | --- |
| (Intercept) | 5.39 | 1.09 | 4.97 | 0.00 |  |
| MFQ Authority | 0.22 | 0.13 | 1.67 | 0.09 | 2.61 |
| MFQ Fairness | -0.63 | 0.17 | -3.69 | **0.00***** | 1.41 |
| MFQ Harm | -0.03 | 0.16 | -0.20 | 0.84 | 1.22 |
| MFQ Ingroup | -0.23 | 0.13 | -1.78 | 0.08 | 2.31 |
| MFQ Purity | 0.02 | 0.12 | 0.14 | 0.89 | 2.29 |
| SDO7 Dominance | -0.16 | 0.12 | -1.41 | 0.16 | 2.54 |
| SDO7 Anti-Egalitarian | -0.05 | 0.11 | -0.48 | 0.63 | 2.34 |

##### Charity Dishonest

Table S10. Poisson regression on the effect of psychological traits on the number of reported doubles. *n*=36, *χ*²(7) = 32.16, *p* = 0.00, *Pseudo-R*² (Cragg-Uhler) = 0.59, *Pseudo-R*² (McFadden) = 0.16, *AIC* = 180.87, *BIC* = 193.53

| **Variables** | **Est.** | **S.E.** | ***z* val.** | ***p*** | ***VIF*** |
| --- | --- | --- | --- | --- | --- |
| (Intercept) | 2.21 | 0.88 | 2.52 | 0.01 |  |
| MFQ Authority | 0.23 | 0.16 | 1.44 | 0.15 | 2.49 |
| MFQ Fairness | 0.49 | 0.19 | 2.57 | **0.01**** | 2.41 |
| MFQ Harm | -0.64 | 0.16 | -3.98 | **0.00***** | 2.19 |
| MFQ Ingroup | 0.20 | 0.16 | 1.26 | 0.21 | 2.22 |
| MFQ Purity | -0.25 | 0.10 | -2.58 | 0.01 | 2.39 |
| SDO7 Dominance | 0.19 | 0.12 | 1.58 | 0.11 | 1.92 |
| SDO7 Anti-Egalitarian | -0.36 | 0.13 | -2.78 | **0.01**** | 1.53 |

#### Poisson regression model with interaction

Anther Poisson regression was carried out to study the effect of psychological traits and the conditions on the cheating behaviour. The dependent variable was the number of reported doubles as before. The explanatory variables were the psychological traits measured by the MFQ and SDO7 questionnaires’ subscales, the conditions (SH vs SD, CH, CD) and their interactions.

To test for multicollinearity, the adjusted generalised variance-inflation factors (GVIF) were calculated, using the *vif* function from the *car* R package. An adjusted GVIF around 1 indicates no correlation with the other predictors, an adjusted GVIF under 2.25 indicates a moderate multicollinearity, while an adjusted GVIF higher than 5 indicates high multicollinearity. Due to high multicollinearity, we rejected this model (see Table S11).

Table S11. Poisson regression on the effect of psychological traits on the number of reported doubles. *n*=144, *χ*²(31) = 112.52, *p* = 0.00, *Pseudo-R*² (Cragg-Uhler) = 0.54, *Pseudo-R*² (McFadden) = 0.15, *AIC* = 724.96, *BIC* = 819.99

| **Variables** | | **Est.** | **S.E.** | ***z* val.** | ***p*** | **adjusted *GVIF*** |
| --- | --- | --- | --- | --- | --- | --- |
| (Intercept) | 0.80 | | 1.43 | 0.56 | 0.57 |  |
| SD | 1.29 | | 1.85 | 0.70 | 0.49 | 14.88 |
| CH | 4.58 | | 1.80 | 2.55 | **0.01**** |  |
| CD | 1.40 | | 1.68 | 0.84 | 0.40 |  |
| MFQ Authority | 0.36 | | 0.18 | 2.07 | **0.04*** | 3.48 |
| MFQ Fairness | 0.33 | | 0.25 | 1.32 | 0.19 | 3.88 |
| MFQ Harm | -0.16 | | 0.19 | -0.84 | 0.40 | 2.86 |
| MFQ Ingroup | -0.09 | | 0.17 | -0.50 | 0.62 | 3.36 |
| MFQ Purity | -0.09 | | 0.13 | -0.73 | 0.46 | 3.26 |
| SDO7 Dominance | -0.07 | | 0.17 | -0.45 | 0.65 | 4.18 |
| SDO7 Anti-Egalitarian | -0.22 | | 0.16 | -1.37 | 0.17 | 4.15 |
| SD * MFQ Authority | -0.44 | | 0.25 | -1.78 | 0.07. | 7.94 |
| CH * MFQ Authority | -0.14 | | 0.22 | -0.64 | 0.52 |  |
| CD * MFQ Authority | -0.13 | | 0.24 | -0.54 | 0.59 |  |
| SD * MFQ Fairness | -0.71 | | 0.29 | -2.47 | **0.01**** | 11.49 |
| CH * MFQ Fairness | -0.96 | | 0.30 | -3.16 | **0.00***** |  |
| CD * MFQ Fairness | 0.16 | | 0.32 | 0.50 | 0.62 |  |
| SD * MFQ Harm | 0.03 | | 0.27 | 0.13 | 0.90 | 10.53 |
| CH * MFQ Harm | 0.13 | | 0.25 | 0.50 | 0.61 |  |
| CH * MFQ Harm | -0.48 | | 0.25 | -1.96 | 0.05. |  |
| SD * MFQ Ingroup | 0.37 | | 0.22 | 1.66 | 0.10 | 7.47 |
| CH * MFQ Ingroup | -0.15 | | 0.22 | -0.67 | 0.50 |  |
| CD * MFQ Ingroup | 0.29 | | 0.24 | 1.22 | 0.22 |  |
| SD * MFQ Purity | 0.33 | | 0.17 | 1.90 | 0.06. | 5.59 |
| CH * MFQ Purity | 0.11 | | 0.17 | 0.63 | 0.53 |  |
| CD * MFQ Purity | -0.16 | | 0.16 | -1.02 | 0.31 |  |
| SD * SDO7 Dominance | 0.11 | | 0.21 | 0.51 | 0.61 | 5.45 |
| CH * SDO7 Dominance | -0.09 | | 0.20 | -0.43 | 0.67 |  |
| CD * SDO7 Dominance | 0.27 | | 0.21 | 1.30 | 0.19 |  |
| SD * SDO7 Anti-Egalitarian | 0.19 | | 0.19 | 0.96 | 0.34 | 6.04 |
| CH * SDO7 Anti-Egalitarian | 0.16 | | 0.19 | 0.85 | 0.40 |  |
| CD * SDO7 Anti-Egalitarian | -0.14 | | 0.20 | -0.69 | 0.49 |  |

### The effect of the experimenter on the finger ratio

The experimenter seems not to affect finger ratio, as regardless of the use of the experimenter in the models, finger ratio’s effect remains non significant when studying the effect of finger ratio and treatment on the number of reported doubles.

The model without the experimenter

Table S12. The effect of the finger ratio and the treatments on the number on the number of doubles. OLS linear regression. *n*=144, *F*(3,140) = 2.10, *p* = 0.10 *R*² = 0.04 *Adj. R*² = 0.02

| **Variables** | **Est.** | **S.E.** | ***t* val.** | ***p*** |
| --- | --- | --- | --- | --- |
| (Intercept) | 10.46 | 6.48 | 1.61 | 0.11 |
| GameS | -1.39 | 0.59 | -2.34 | 0.02 |
| PartnerH | -0.33 | 0.59 | -0.56 | 0.58 |
| Finger ratio | -4.86 | 6.58 | -0.74 | 0.46 |

The model with the experimenter as explanatory variable

Table S13. The effect of the experimenters, the finger ratio and the treatments on the number on the number of doubles.OLS linear regression. *n*=144, *F*(5,138) = 1.33, *p* = 0.26 *R*² = 0.05 *Adj. R*² = 0.01

| **Variables** | **Est.** | **S.E.** | ***t* val.** | ***p*** |
| --- | --- | --- | --- | --- |
| (Intercept) | 10.16 | 6.74 | 1.51 | 0.13 |
| GameS | -1.44 | 0.60 | -2.40 | 0.02 |
| PartnerH | -0.27 | 0.50 | -0.45 | 0.65 |
| Finger ratio | -4.60 | 6.88 | -0.67 | 0.50 |
| experimenterDD | -0.13 | 0.86 | -0.15 | 0.88 |
| experimenterJM | 0.55 | 0.91 | 0.60 | 0.55 |

The model with the experimenter as random factor

Table S14. The effect of the finger ratio and the treatments on the number on the number of doubles, the experimenter used as random factor

Fixed effects:

| **Variables** | ***numDF*** | ***denDF*** | ***F-*value** | ***p*** |
| --- | --- | --- | --- | --- |
| (Intercept) | 1 | 138 | 266.31 | 0.000 |
| Game | 1 | 138 | 5.404 | 0.022 |
| Partner | 1 | 138 | 0.345 | 0.449 |
| Finger ratio | 1 | 138 | 0.545 | 0.461 |

Random effects: ~ experimenter

|  | **intercept** | **residual** |
| --- | --- | --- |
| Std dev | 0.000 | 3.467 |

### The distribution of the rolled values

#### Values of player A-s

These numbers were generated by the computer. In the Honest treatments, the game simulated an even dice, and therefore we expect its result to come from the uniform distribution.

We performed Chi-square tests to see if the rolled values by the computer come from a uniform distribution. We used *chisq.test* function in R, so we applied a test statistic and P-value based on a Monte Carlo approach. In this case, there is no assumed chi-square distribution for the test statistic, so there is also no df parameter involved. The results are shown in the next table. The computer rolled values show a uniform distribution in the Honest conditions, and a deviation from the uniform distribution in the Dishonest conditions.

Table S15. The result of the Chi-squared test

| **Condition** | ***χ*^2^** | ***p*** |
| --- | --- | --- |
| SH | 0.983 | 0.967 |
| SD | 497.067 | **0.000** |
| CH | 2.217 | 0.810 |
| CD | 534.017 | **0.000** |

#### Values of player B-s

We performed Chi-square tests by using *chisq.test* function in R which applies a test statistic and P-value based on a Monte Carlo approach. In this case, there is no assumed chi-square distribution for the test statistic, so there is also no df parameter involved. The results are shown in the next table.

Reported values in conditions with a dishonest partner (SD and CD) and the SH condition are significantly different from the uniform distribution, probably because participants cheated and matched player As' values, which in turn were sampled from a screwed distribution.

Reported values in conditions CH does not differ significantly from the uniform distributions, however, these results do not show whether participants cheated. The distribution can be uniform because they did not cheat or because they cheated by matching player As' values that in turn were sampled from a uniform distribution.

Table S15. The result of the Chi-squared test

| **Condition** | ***χ*^2^** | ***p*** |
| --- | --- | --- |
| SH | 14.667 | **0.012** |
| SD | 11.233 | **0.036** |
| CH | 3.283 | 0.674 |
| CD | 14.400 | **0.011** |

References

Faragó, L., & Kende, A. (2017). Az elnyomás támogatása vagy az egyenlőség ellenzése? Az új szociális dominancia orientáció skála (SDO7) vizsgálata. Alkalmazott pszichológia, 17:115-135 (in Hungarian).

Graham, J., Nosek, B. A., Haidt, J., Iyer, R., Koleva, S., & Ditto, P. H. (2011). Mapping the moral domain. J of Pers and Soc Psychol, 101: 366. https://doi.org/[10.1037/a0021847](http://dx.doi.org/10.1037/a0021847)

Hadarics, M., & Kende, A. (2017). A closer look at intergroup threat within the dual process model framework: The mediating role of moral foundations. Psychol Thought, 10: 167–177. <https://doi.org/10.5964/psyct.v10i1.210>

Ho, A. K., Sidanius, J., Kteily, N., Sheehy-Skeffington, J., Pratto, F., Henkel, K. E., … and Stewart, A. L. (2015). The nature of social dominance orientation: Theorizing and measuring preferences for intergroup inequality using the new SDO₇ scale. J of Pers and Soc Psychol, *109:* 1003-1028. https://doi.org/[10.1037/pspi0000033](https://doi.org/10.1037/pspi0000033)

Weisel, O., and Shalvi, S. (2015). The collaborative roots of corruption. Proc Natl Acad Sci USA, 112: 10651-10656. <https://doi.org/10.1073/pnas.1423035112>

Wouda, J., Bijlstra, G., Frankenhuis, W. E., Wigboldus, D. H. J. (2017): The Collaborative Roots of Corruption? A Replication of Weisel & Shalvi (2015) Collabra: Psychology, 3, 1, 27: 1–3. <https://www.collabra.org/articles/10.1525/collabra.97>
